# Genome of *Methylomonas* sp. AM2-LC, representing a methanotrophic bacterial species isolated from water column of a boreal, oxygen-stratified lake

**DOI:** 10.3389/fgene.2024.1440435

**Published:** 2024-08-30

**Authors:** Antti J. Rissanen, Rahul Mangayil, Ramita Khanongnuch

**Affiliations:** ^1^ Faculty of Engineering and Natural Sciences, Tampere University, Tampere, Finland; ^2^ Natural Resources Institute Finland, Helsinki, Finland; ^3^ Department of Bioproducts and Biosystems, School of Chemical Engineering, Aalto University, Espoo, Finland; ^4^ Department of Geography, Institute of Ecology and Earth Sciences, University of Tartu, Tartu, Estonia

**Keywords:** methanotroph, greenhouse gas, methane bioconversion, Methylomonas, biogas, natural gas, climate change, lake

## 1 Introduction

Methanotrophic bacteria are a special group of bacteria that consume methane as their energy and carbon source. They are roughly divided into aerobic gammaproteobacterial, alphaproteobacterial and verrucomicrobial methanotrophs (that use O_2_ as their primary electron acceptor), and anaerobic bacteria of genus *Candidatus* Methylomirabilis (that use nitrite to oxidize methane) (Guerrero-Cruz et al., 2021). Methanotrophic bacteria play a crucial role in reducing the methane emissions from natural methanogenic ecosystems, like rivers, lakes and wetlands (Hanson and Hanson, 1996). They are also actively harnessed for their biotechnological potential to mitigate methane emissions from anthropogenic ecosystems (e.g., biofilters at landfills) and to convert methane in biogas and natural gas into various value-added products (e.g., single-cell protein and bioplastics) (Strong et al., 2015).

Comparative genomics of bacteria form the basis of the current bacterial taxonomy, like that of methanotrophic bacteria (Orata et al., 2018). In addition to comparative analysis, genome sequences of methanotroph isolates provide an important backbone database for taxonomic and functional analysis of the vast and constantly increasing shotgun metagenomic data, especially metagenome-assembled genomes (MAG) of putative methanotrophs, collected from environment (Buck et al., 2021; Khanongnuch et al., 2023). Furthermore, genomic data provide crucial insights into potentially novel and testable metabolisms in methanotrophic bacteria, relevant both to the understanding of environmental methane cycling and for biotechnological applications, such as fermentation, i.e., conversion of methane to organic acids and H_2_ (Kalyuzhnaya et al., 2013), denitrification (Kits et al., 2015), extracellular electron transfer (Zheng et al., 2020), and oxidation of alternative electron donors (Gwak et al., 2022). Genetic engineering of methanotrophs to enhance their methane consumption and bioconversion efficiencies, and to increase the range of methane-derived products, also benefit greatly from the genomic data (Henard and Guarnieri, 2018; Jeong et al., 2023).

Here, we report the genome sequence of a strain *Methylomonas* sp. AM2-LC, which was isolated from the water column of a boreal, humic, O_2_-stratified lake, located in Southern Finland. It represents a putatively novel species of *Methylomonas* sp., a methanotroph genus widely present in various methanogenic ecosystems (Bussmann et al., 2021; Danilova et al., 2013; Hoefman et al., 2014; Ogiso et al., 2012; Zhu et al., 2020), and an attractive methane bioconversion candidate (Patel et al., 2018; Patel et al., 2022; Tikhonova et al., 2023).

## 2 Value of the data

The genome of *Methylomonas* sp. AM2-LC can be used as a valuable resource to conduct comparative functional and taxonomic analyses among methanotroph isolates and environmental MAGs representing methanotrophic bacteria, especially *Methylomonas* sp. It will aid in refining the taxonomy of *Methylomonas* sp. and in enhancing the understanding of the metabolic capabilities of methanotrophs and their distribution in environment. More specifically, the genome can be explored for putative novel functions having biogeochemical and/or biotechnological interest, and to design genetic engineering experiments.

## 3 Materials and methods

### 3.1 Strain isolation and DNA extraction

The strain AM2-LC was isolated from water samples collected at 4.5 m depth of Lake Alinen Mustajärvi, Southern Finland, on 27 September 2022. The temperature, pH and concentrations of O_2_, NH_4_^+^-N, NO_3_^−^ + NO_2_^−^-N and PO_4_^3-^-P at the time of sampling were 5.3°C, 5.54, 0.41 mg/L, 335 μg/L, <5 μg/L and 4 μg/L. When the lake water arrived at the laboratory, pre-enrichment was conducted by transferring 100 mL of the lake water to 500 mL sterile glass bottles sealed with septum and screw cap, and the headspace was replaced with 20% CH_4_. After ∼30 days, the pre-enriched lake water was used as an inoculum to further enrich methanotrophs. This was done by diluting the pre-enriched lake water 1:10 in the modified ammonium mineral salts (AMS) medium. The latter consists of (g/L): NH_4_Cl (0.03), MgSO_4_·7H_2_O (1), CaCl_2_·2H_2_O (0.2), phosphate buffer containing K_2_HPO_4_ (0.28), KH_2_PO_4_ (0.33), pH 6.6, iron (III)-EDTA (0.004), Na_2_MoO_4_·2H_2_O (0.00023), 0.1% (*v*/*v*) of trace element containing (g/L): CuSO_4_·5H_2_O (1), FeSO_4_·7H_2_O (0.5), ZnSO_4_·7H_2_O (0.4), H_3_BO_3_ (0.015), CoCl_2_·6H_2_O (0.05), EDTA-Na_2_ (0.25), MnCl·4H_2_O (0.02), NiCl_2_·6H_2_O (0.01), and 0.5% (*v/v*) vitamin solution containing (mg/L), pyridoxine hydrochloride (10), thiamine-HCl (5), riboflavin (5), nicotinic acid (5), thioctic acid (5), folic acid (2). Additionally, 1 µM La_2_O_3_ and CeCl_3_·7H_2_O were supplemented to the modified AMS medium. To inhibit fungal growth, 2.5 μg/mL of amphotericin B solution (Sigma-Aldrich Ltd.) was added to the modified AMS medium. The enrichment was conducted in serum bottles filled with ∼8% (*v/v*) of the modified AMS medium, and CH_4_, sterilized with 0.22 µm sterile syringe filter, was added to the headspace to obtain a 20:80 ratio CH_4_ and air. When the turbidity was observed, the enriched culture was transferred into a new bottle with fresh medium and headspace gas replenishment. The serial dilutions of 1:10^6^ was performed until a pure culture was obtained. The culture purity was verified by streaking onto nutrient-rich agar (5 g/L tryptone, 2.5 g/L yeast extract, 1 g/L glucose, and 20 g/L agar) showing no growth and observing by a light microscope. So far, the strain AM2-LC could grow at pH 6.0, 6.6, and 6.8 in a cold room (5°C ± 1.5 °C) and room temperature (20°C ± 2 °C). The growth was not observed in a liquid medium when ammonium was replaced with nitrate as a nitrogen source. The isolate morphology was observed by microscopy as rod shape (0.9–2.5 µm in length and 0.8–1.3 µm in width) (Supplementary Figure S1 in Supplementary Material S1). Furthermore, the strain was able to grow with 2% CH_4_ in the headspace (Supplementary Figure S2C in Supplementary Material S1). The culture is available at the laboratory at Tampere University, Finland. To preserve it, the cell pellets were resuspended in 1XPBS (pH 7.4) containing 7% DMSO and stored at −80 °C.

Genomic DNA was extracted using GeneJET Genomic DNA Purification Kit and quantified using a Qubit 3.0 Fluorometer and a dsDNA HS Assay Kit (Thermo Fisher Scientific, Waltham, MA, United States).

### 3.2 16S rRNA gene sequencing and phylogenetic analysis

Using the identification service offered by Macrogen (Amsterdam, Netherlands), the 16S rRNA genes of the strain AM2-LC were amplified from the genomic DNA (gDNA) using primers 27F (AGAGTTTGATCMTGGCTCAG) and 1492R (TACGGYTACCTTGTTACGACTT) and sequenced using primer pairs 785F (GGA​TTA​GAT​ACC​CTG​GTA) and 907R (CCGTCAATTCMTTTRAGTTT). The 16S rRNA gene sequence alignment with reference sequences (SINA aligner v.1.2.12) and the phylogenetic tree analysis (FastTree v. 2.1.11, ML model: Generalized Time-Reversible) was performed using the Silva Alignment, Classification and Tree Service (Pruesse et al., 2012).

### 3.3 Genome sequencing and analysis

The gDNA sequencing, including library preparation and sequencing of both long (PacBio SMRT, CCS sequencing mode, PacBio Revio) and short reads [Illumina NovaSeq X (PE150)] was performed as a service provided by Biomarker Technologies (BMK) GmbH. The sequencing facility also provided bioinformatic services such as filtering of long (reads with length <2 kb removed) and short reads [fastp v0.23.2, (Chen et al., 2018), to remove adapter and low quality reads], genome assembly [Hifiasm v. 0.14, (Cheng et al., 2021; Cheng et al., 2022)], genome assembly improvement [Pilon v 1.22, (Walker et al., 2014)], and genome cyclizing [circlator v. 1.5.5, (Hunt et al., 2015)]. The genome completeness and contamination was assessed using checkM (v1.2.2, Methylococcales. ms marker set) (Parks et al., 2015).

The protein sequences, repetitive sequences, tRNAs, rRNAs, CRISPR regions, genomic islands, prophages, and biosynthetic gene clusters (BGC) were predicted using Prodigal (v. 2.6) (Hyatt et al., 2010), RepeatMasker (v4.0.5) (Tarailo-Graovac and Chen, 2009), tRNAscan-SE (v2.0) (Chan and Lowe, 2019), Infernal (v1.1.3) (Nawrocki and Eddy, 2013), CRT (v1.2) (Bland et al., 2007), IslandPath-DIMOB (v0.2) (Bertelli and Brinkman, 2018), PhiSpy (v2.3) (Akhter et al., 2012), and antiSMASH (v5.0.0) (Blin et al., 2019), respectively. The genome-wide phylogenetic tree was built from protein alignments generated in PhyloPhlAn (v. 3.0.67; PhyloPhlAn database including 400 universal marker genes and “-diversity low” - argument) (Segata et al., 2013; Asnicar et al., 2020) using the maximum-likelihood algorithm (PROTCATLG − model) with 100 bootstrap replicates in RAxML (v. 8.2.12) (Stamatakis, 2014). Average nucleotide identities (ANI) with reference genomes were calculated using ANI calculator (http://enve-omics.ce.gatech.edu/ani/, accessed on April 2024) (Goris et al., 2007). Digital DNA-DNA hybridization (dDDH) comparisons with reference genomes were done using the Type Strain Genome Server (TYGS) online service (https://tygs.dsmz.de/, accessed on April 2024) (Meier-Kolthoff and Göker, 2019). The functional annotation was performed via BLAST search against NCBI´s Nr, eggNOG (Powell et al., 2014), GO (Ashburner et al., 2000), Pfam (Finn et al., 2014), SwissProt and TrEMBL databases (Bairoch and Apweiler, 1996). KofamKOALA (https://www.genome.jp/tools/kofamkoala/, accessed on April 2024) was used to search the predicted genes against the KEGG database (Kanehisa et al., 2004; Aramaki et al., 2020).

### 3.4 Preliminary data analysis

The statistics of *de novo* assembly and genome characteristics are reported in Table 1. The genome, with full length of 5394081 bp and G + C content of 42.7%, consisted of three contigs of which one was the chromosome (4971665 bp) and two were plasmids (288757 bp and 133659 bp) (Supplementary Figure S3 in Supplementary Material S1). The genome was of very high quality as judged by the high completeness and low contamination estimates (Table 1) (Bowers et al., 2017). Furthermore, the genome contained 4,933 coding sequences, 9 rRNA and 48 tRNA genes, 14 CRISPR regions, 24 genomic island regions, 10 prophages, 6 biosynthetic gene clusters, and had 6,780 bp of repetitive sequences (Table 1). See further results on the analysis of these genome characteristics (including predicted protein sequences) in Supplementary Material S2.

**TABLE 1:** Statistics of *de novo* genome assembly, genome characteristics and taxonomically closest reference strain of *Methylomonas* sp. AM2-LC.

Feature	Strain AM2-LC
Total sequence length (bp)	5394081
Number of contigs	3 (1 chromosome +2 plasmids)
Chromosome length (bp)	4971665
plasmid 1 length (bp)	288757
plasmid 2 length (bp)	133659
N50 (bp)	4971665
G + C - content (%)	42.7%
Number of coding sequences (CDS)	4,933
Repetitive sequence length (bp)	6,780 (0.13%)
Number of 5S, 16S and 23S rRNA genes	3 (5S), 3 (16S), 3 (23S)
Number of tRNA genes	48
Number of CRISPR	14
Number of genomic islands	24
Number of prophages	10
Number of biosynthetic gene clusters	6
Genome completeness (%)	99.2
Genome contamination (%)	2.0
Closest reference: *Methylomonas paludis* with	
16S rRNA gene identity - %	98.3% (HE801216.1)
ANI - %	77.9% (GCA_018734325.1)
dDDH - %	21.3% (GCA_018734325.1)

The strain AM2-LC was most closely related with *M. paludis* (Danilova et al., 2013; Rissanen et al., 2021) with 98.3% similarity in the 16S rRNA gene comparisons and with 77.9% ANI and 21.3% dDDH in the genome-level comparisons (Table 1). It also positioned closest to *M. paludis* in phylogenetic and phylogenomic tree analyses (Supplementary Figure S4 in Supplementary Material S1). The respective similarities with representatives of *Methylomonas* sp. generally varied (min-max) 94.3%–98.3% (16S rRNA genes), 76.3%–77.9% (ANI) and 18.6%–21.3% (dDDH). Given these being below the widely used thresholds to delineate unique species, 98.65%, 95%, and 70%, for 16S rRNA genes, ANI, and dDDH, respectively (Goris et al., 2007; Auch et al., 2010; Meier-Kolthoff et al., 2013; Kim et al., 2014; Chun et al., 2018; Orata et al., 2018), the strain AM2-LC very likely represents a novel species of genus *Methylomonas*.

**FIGURE 1:**
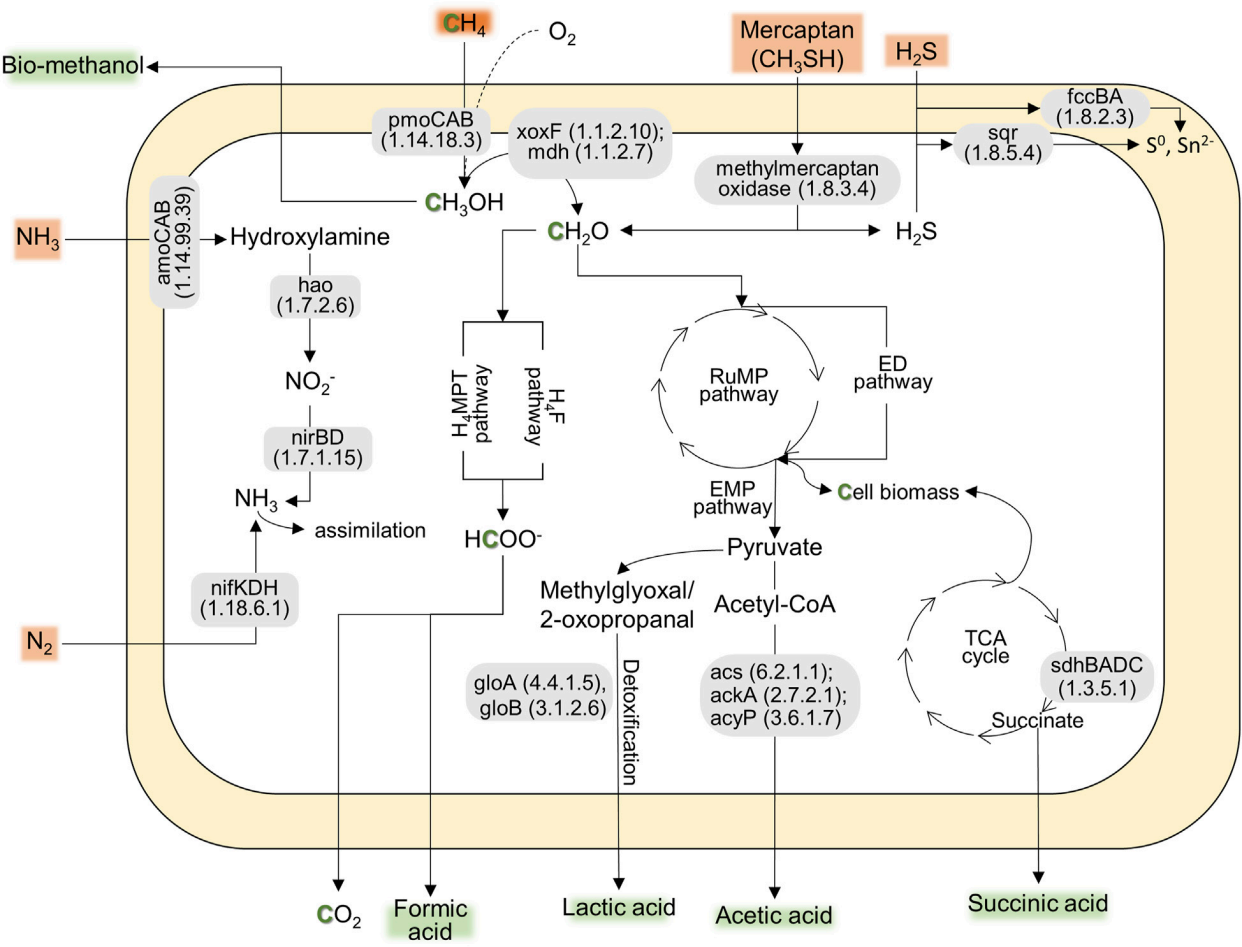
The predicted pathways indicating the genetic potential of AM2-LC for CH_4_ conversion into CH_4_-derived compounds (e.g., methanol and organic acids) and nitrogen fixation, contributing to the ecosystem function and bioconversion of CH_4_. Additionally, the strain shows genetic potential for tolerating toxic compounds commonly found in biogas, including genes for converting hydrogen sulfide, mercaptan, and NH_3_. The genes present in the pathway are based on KofamKOALA (KEGG Orthology and Function Annotation by KEGG Orthology And Links Annotation) (Supplementary Material S3).

According to the preliminary annotation analysis (Figure 1), the strain AM2-LC’s genome contained particulate methane monooxygenases (pmoCAB) for methane conversion into methanol, while soluble methane monooxygenases (mmoXYBZDC) were absent. In addition, the strain encoded the pxm operon (pxmABC), i.e., a copper membrane monooxygenase of unknown function (Tavormina et al., 2011). The genome contained both calcium- (mxaLKCAIGJFD) and lanthanide-dependent (xoxF) methanol dehydrogenases for converting methanol to formaldehyde. Genes involved in tetrahydromethanopterin (H4MPT)-mediated pathway, catalyzing the conversion of formaldehyde into formate, were also present in the genome. The genome also contained genes encoding the RuMP pathway [for carbon (formaldehyde) assimilation], the oxidative TCA cycle, and Entner-Doudoroff and Embden-Meyerhof-Parnas pathways for energy conservation (Figure 1). Similar to the closely related strain *M. paludis* S2AM, the strain AM2-LC can potentially convert methane into industrially important organic acids, i.e., formic acid, lactic acid, acetic acid, and succinic acid (Figure 1) (Khanongnuch et al., 2023; Strong et al., 2015). Furthermore, the genome included genes encoding N_2_ fixation (nitrogenase, nifKDH), assimilation of nitrate (nitrate reductase, nasA and nitrite reductase, nirDB), oxidation of hydroxylamine into nitrite (hydroxylamine dehydrogenase, hao). Interestingly, the strain contains the genetic potential to oxidize other toxic compounds commonly found in biogas, including the conversion of hydrogen sulfide to sulfur (sulfide dehydrogenase, fccBA; sulfide-quinone oxidoreductase, sqr) and conversion of methylmercaptan into formaldehyde and hydrogen sulfide (methylmercaptan (MM)-oxidase) (Figure 1). The strain is likely incapable of carotenoid biosynthesis as lacking the relevant functional genes. See the detailed results on the functional annotation analyses in Supplementary Material S3. The results of COG annotation analysis are also visualized in Supplementary Figure S3 in Supplementary Material S1.

## Data Availability

All data presented in this study is publicly available. The raw sequence data is deposited in NCBI’s SRA under the Bioproject PRJNA1112122 and Biosample SAMN41414985. The assembled genome has been deposited at NCBI’s Genbank under accession number CP157005 for chromosome, CP157006 for plasmid 1, and CP157007 for plasmid 2. The independently sequenced 16S rRNA gene sequence is deposited in Genbank under accession number PP800278. Data on genome characteristics (incl. predicted protein sequences) and functional annotation analyses are found in Supplementary Material S2, S3.
