# Supplementary material for: Genome of Methylomonas sp. AM2-LC, representing a methanotrophic bacterial species isolated from water column of a boreal, oxygen-stratified lake
Source: Front Genet. 2024 Aug 30;15:1440435. doi: 10.3389/fgene.2024.1440435 (PMC11392852; doi:10.3389/fgene.2024.1440435)
Supplement: Supplementary file 1 [file DataSheet3.ZIP › Supplementary_File_3_rev/02.nr_annotation/nr.anno_species_stat.pdf]

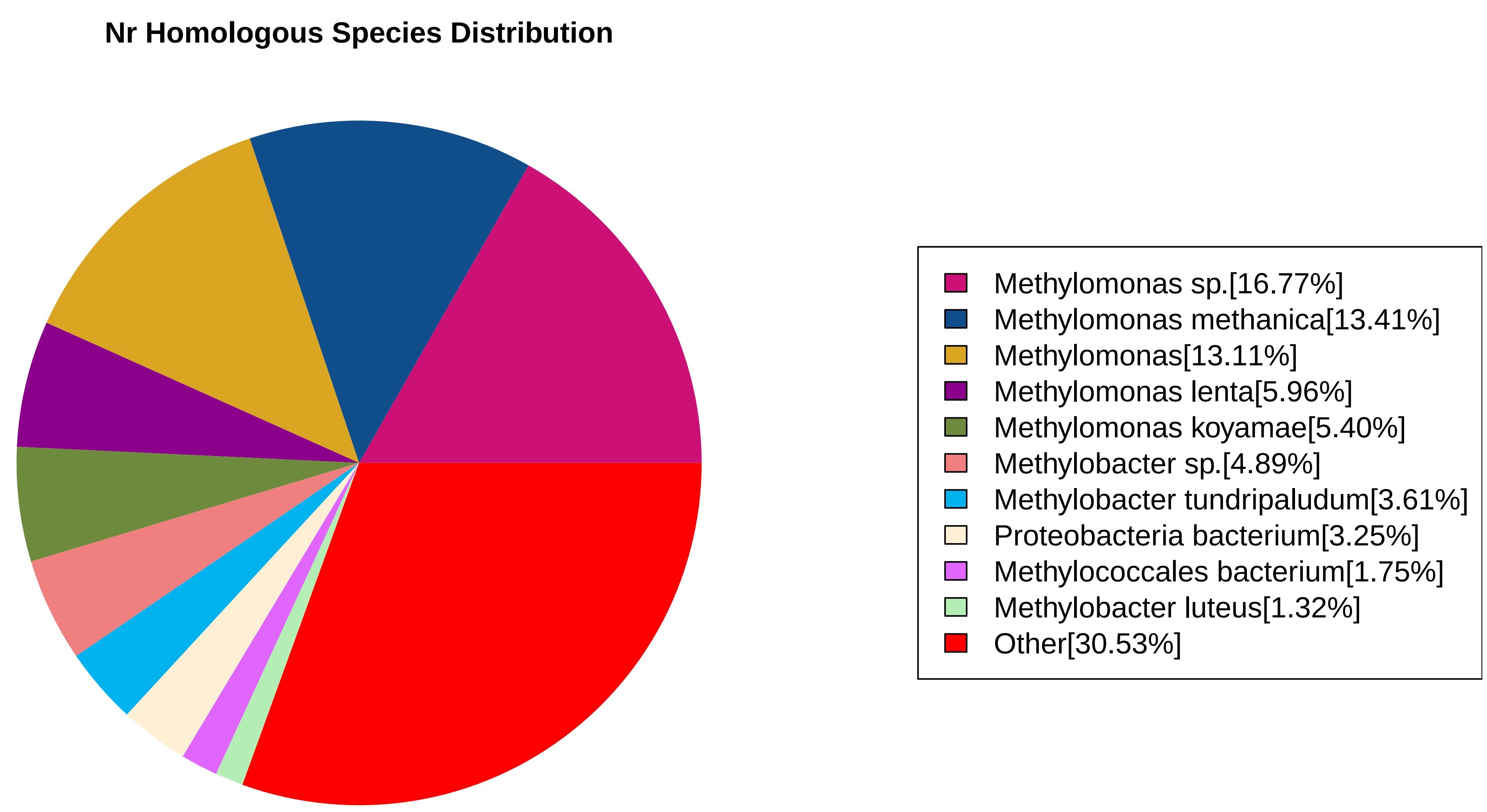
Nr Homologous Species Distribution
Methylomonas sp.[16.77%]
Methylomonas methanica[13.41%]
Methylomonas[13.11%]
Methylomonas lenta[5.96%]
Methylomonas koyamae[5.40%]
Methylobacter sp.[4.89%]
Methylobacter tundripaludum[3.61%]
Proteobacteria bacterium[3.25%]
Methylococcales bacterium[1.75%]
Methylobacter luteus[1.32%]
Other[30.53%]
