# Supplementary figures and images for: Genome of Methylomonas sp. AM2-LC, representing a methanotrophic bacterial species isolated from water column of a boreal, oxygen-stratified lake
Source: Front Genet. 2024 Aug 30;15:1440435. doi: 10.3389/fgene.2024.1440435 (PMC11392852; doi:10.3389/fgene.2024.1440435)

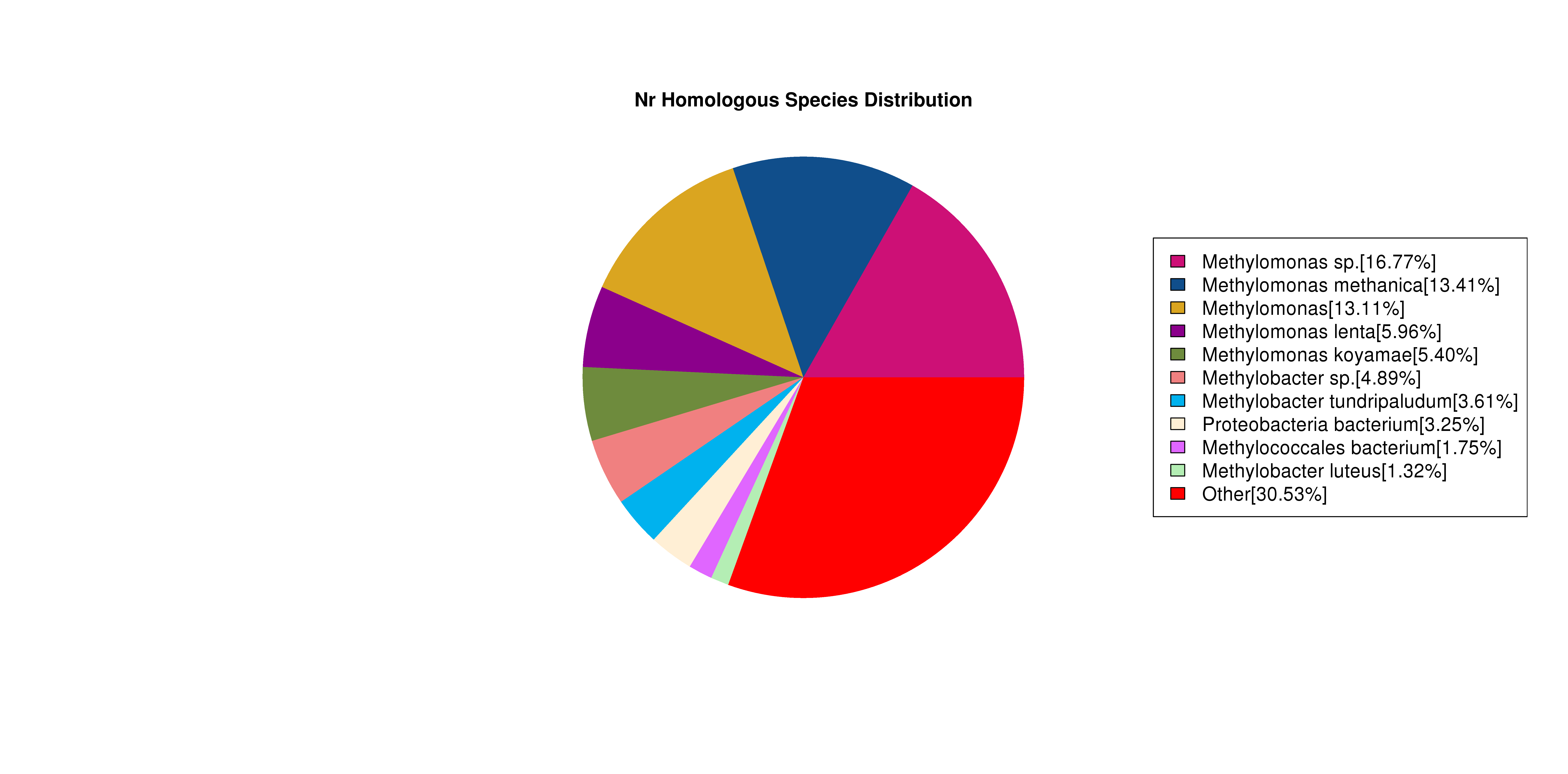

Supplement: Supplementary file 1 [file DataSheet3.ZIP › Supplementary_File_3_rev/02.nr_annotation/nr.anno_species_stat.png]

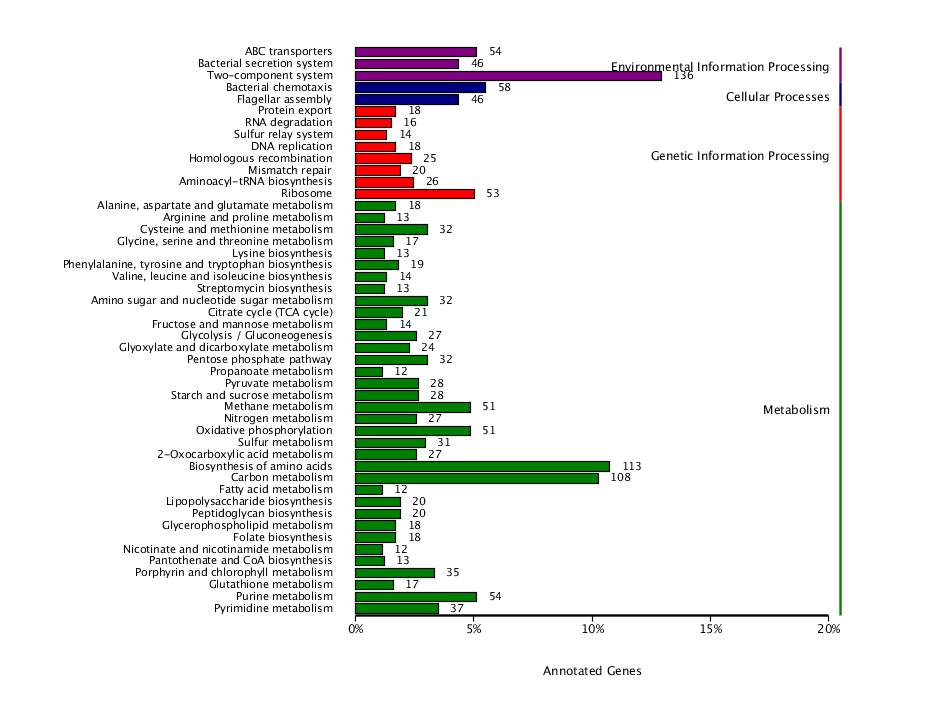

Supplement: Supplementary file 1 [file DataSheet3.ZIP › Supplementary_File_3_rev/03.kegg_annotation/kegg.pathway.png]

# eggNOG Function Classification of Consensus Sequence

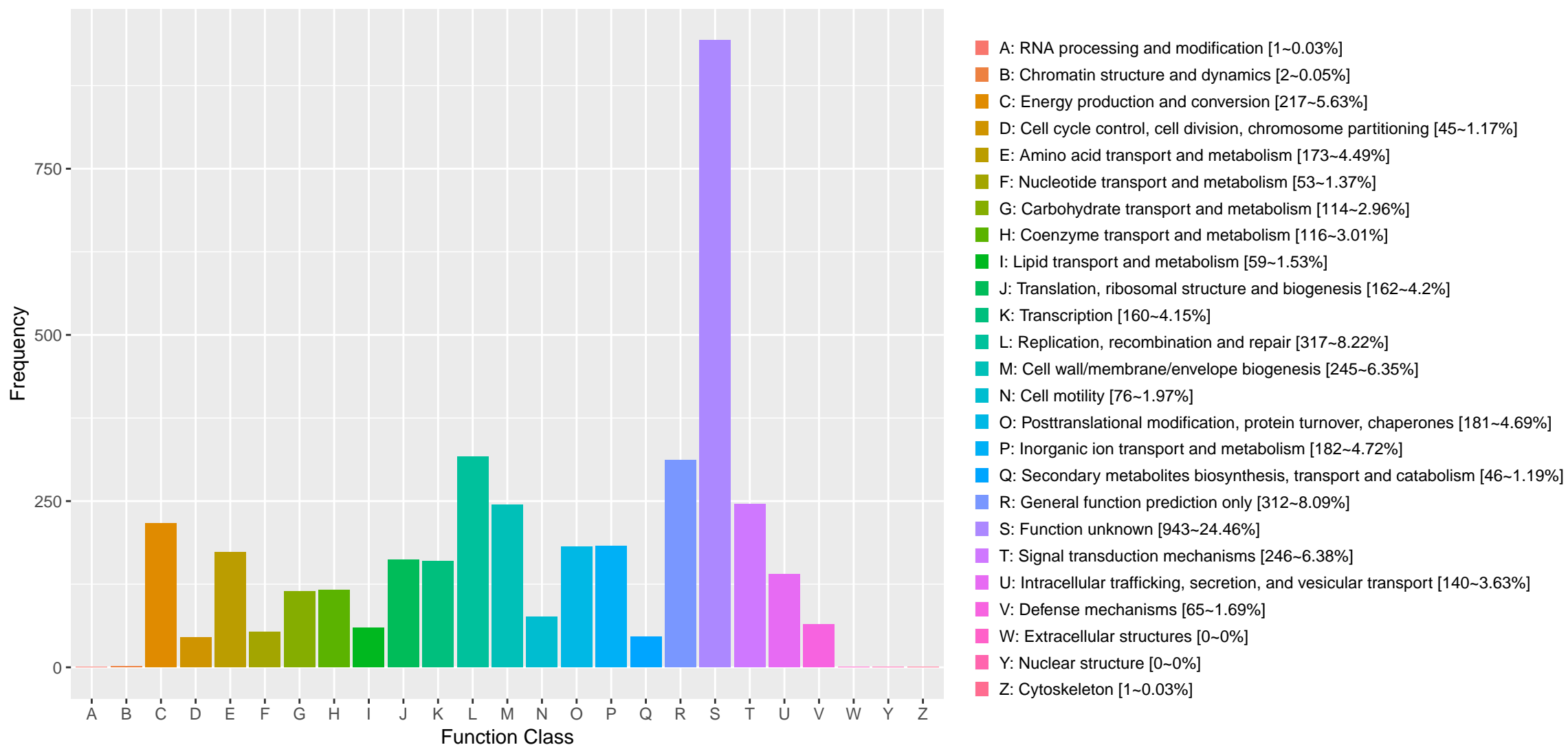

Supplement: Supplementary file 1 [file DataSheet3.ZIP › Supplementary_File_3_rev/04.eggNOG_annotation/eggNOG.Class.stat.pdf]

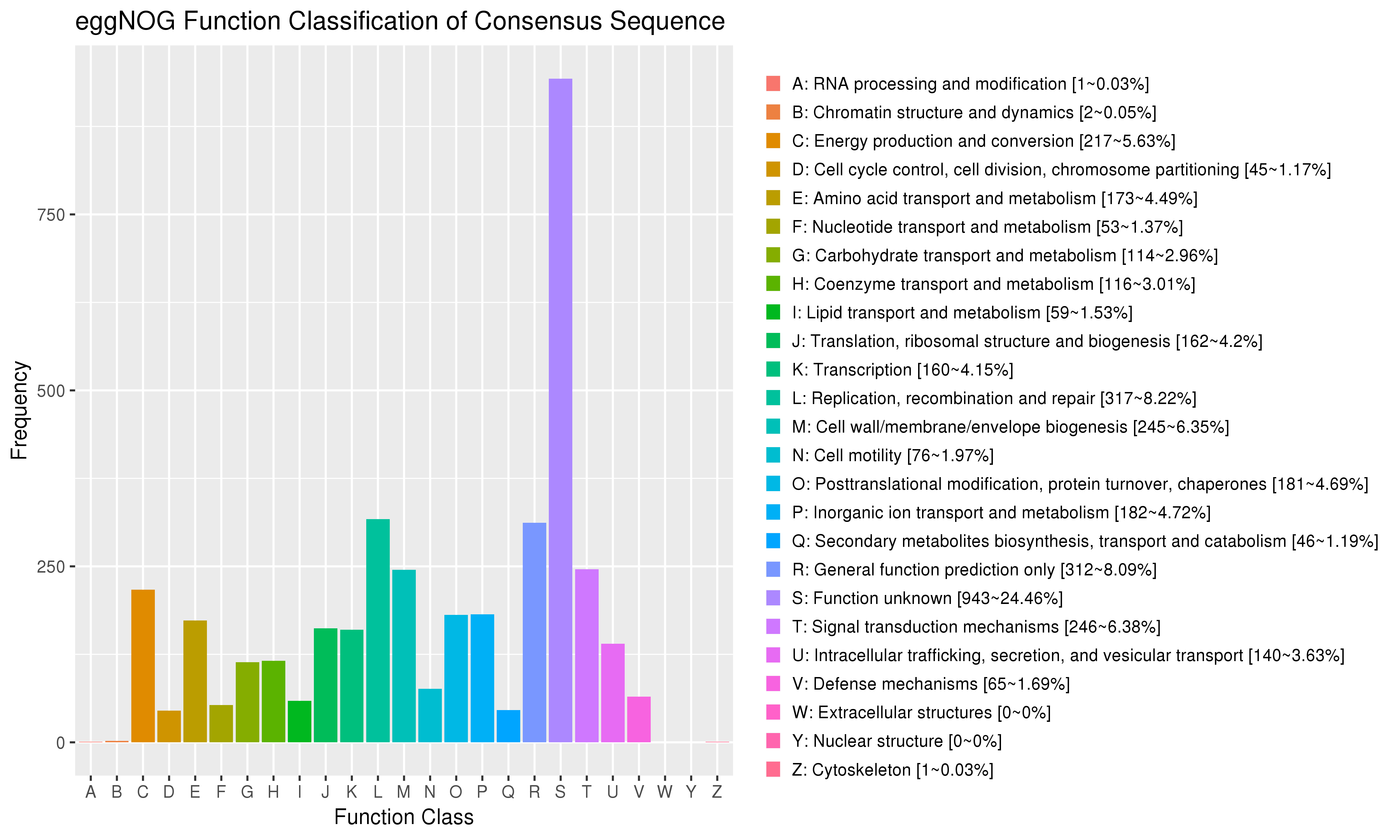

Supplement: Supplementary file 1 [file DataSheet3.ZIP › Supplementary_File_3_rev/04.eggNOG_annotation/eggNOG.Class.stat.png]

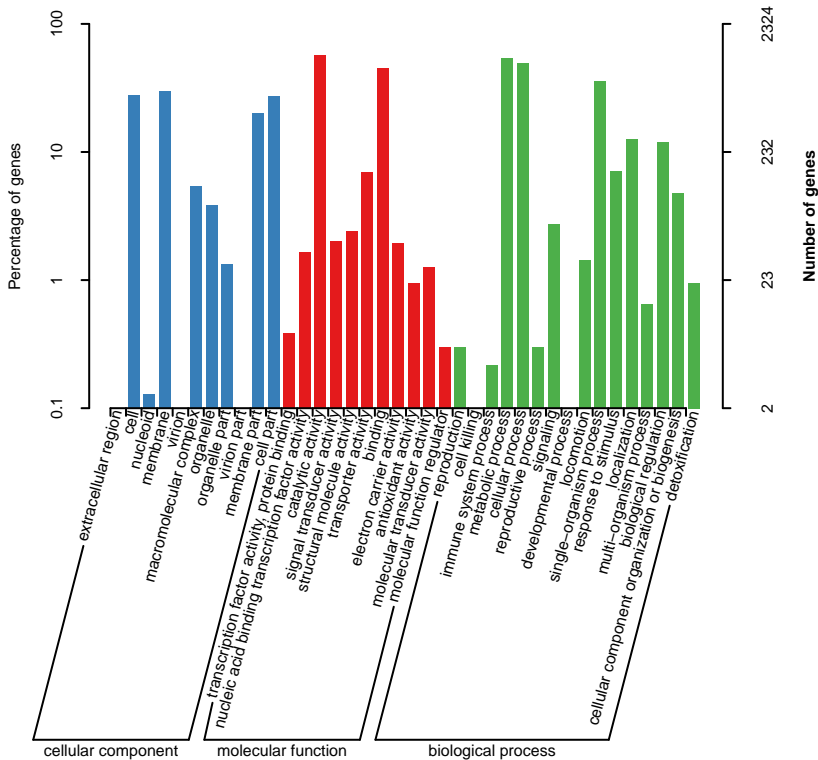

Supplement: Supplementary file 1 [file DataSheet3.ZIP › Supplementary_File_3_rev/05.GO_annotation/GO.second_level.stat.pdf]

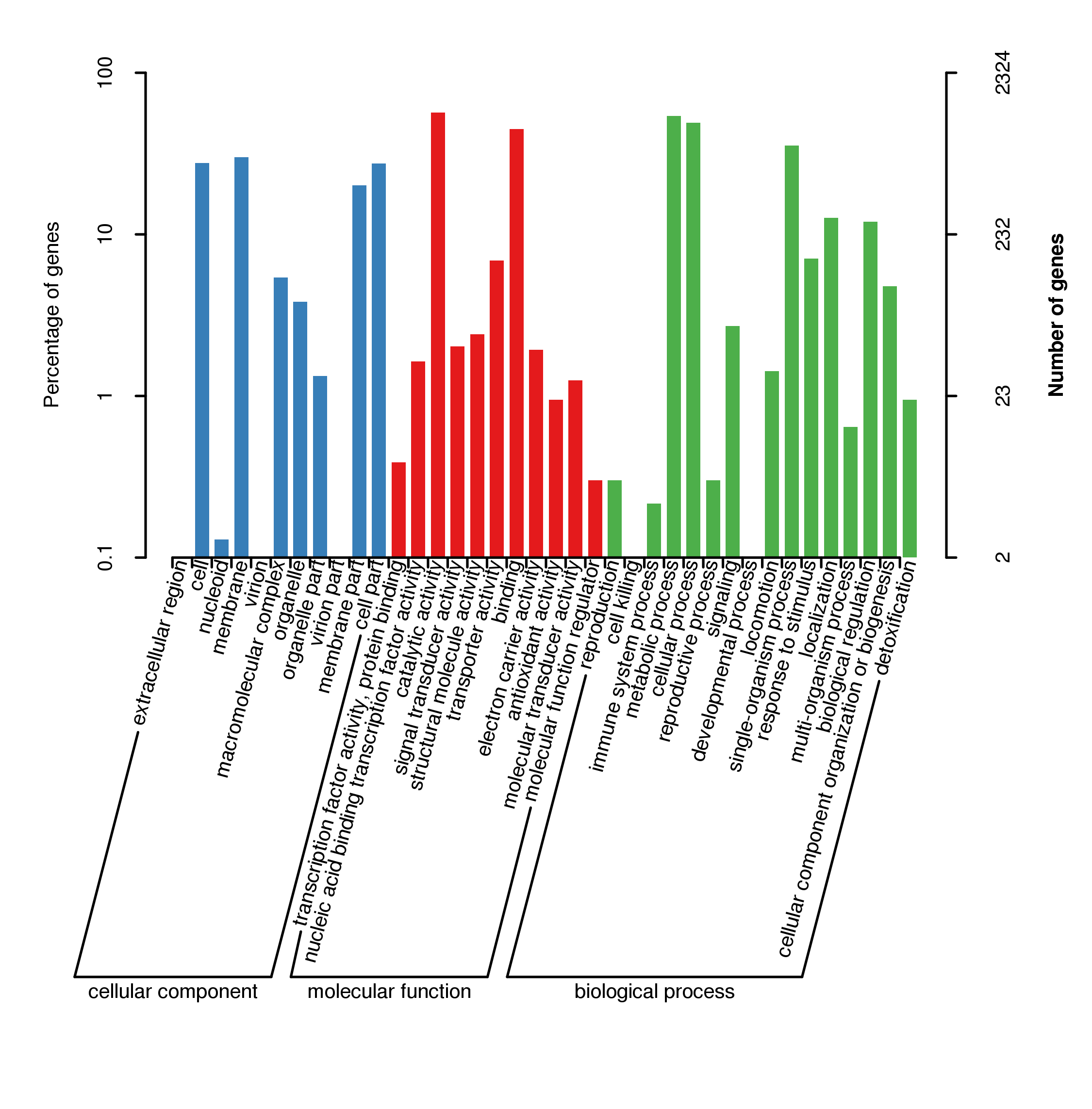

Supplement: Supplementary file 1 [file DataSheet3.ZIP › Supplementary_File_3_rev/05.GO_annotation/GO.second_level.stat.png]

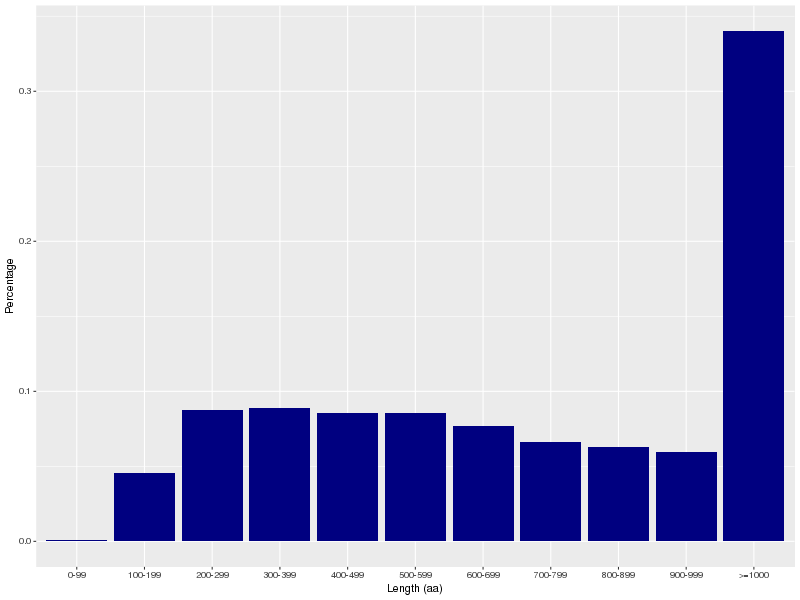

Supplement: Supplementary file 3 [file DataSheet2.ZIP › Supplementary_File_2/01.genomic_gene/protein_length_distribution.png]

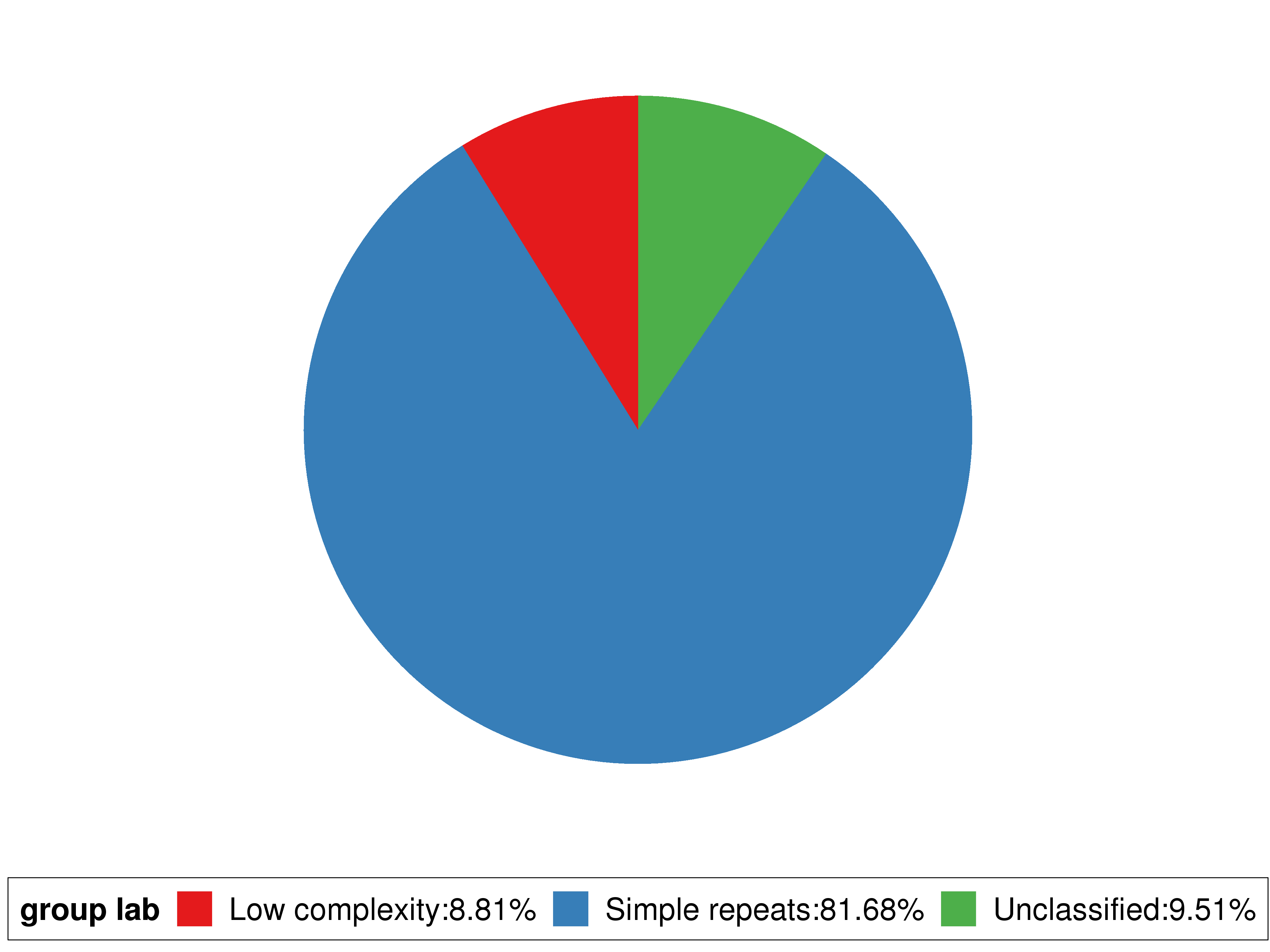

Supplement: Supplementary file 3 [file DataSheet2.ZIP › Supplementary_File_2/02.repetitive_sequences/repetitive_sequence_class_pie.png]
