# Supplementary material for: Genome of Methylomonas sp. AM2-LC, representing a methanotrophic bacterial species isolated from water column of a boreal, oxygen-stratified lake
Source: Front Genet. 2024 Aug 30;15:1440435. doi: 10.3389/fgene.2024.1440435 (PMC11392852; doi:10.3389/fgene.2024.1440435)
Supplement: Supplementary file 2 [file DataSheet1.PDF]

Supplementary File 1:

Supplementary Figures S1-S4

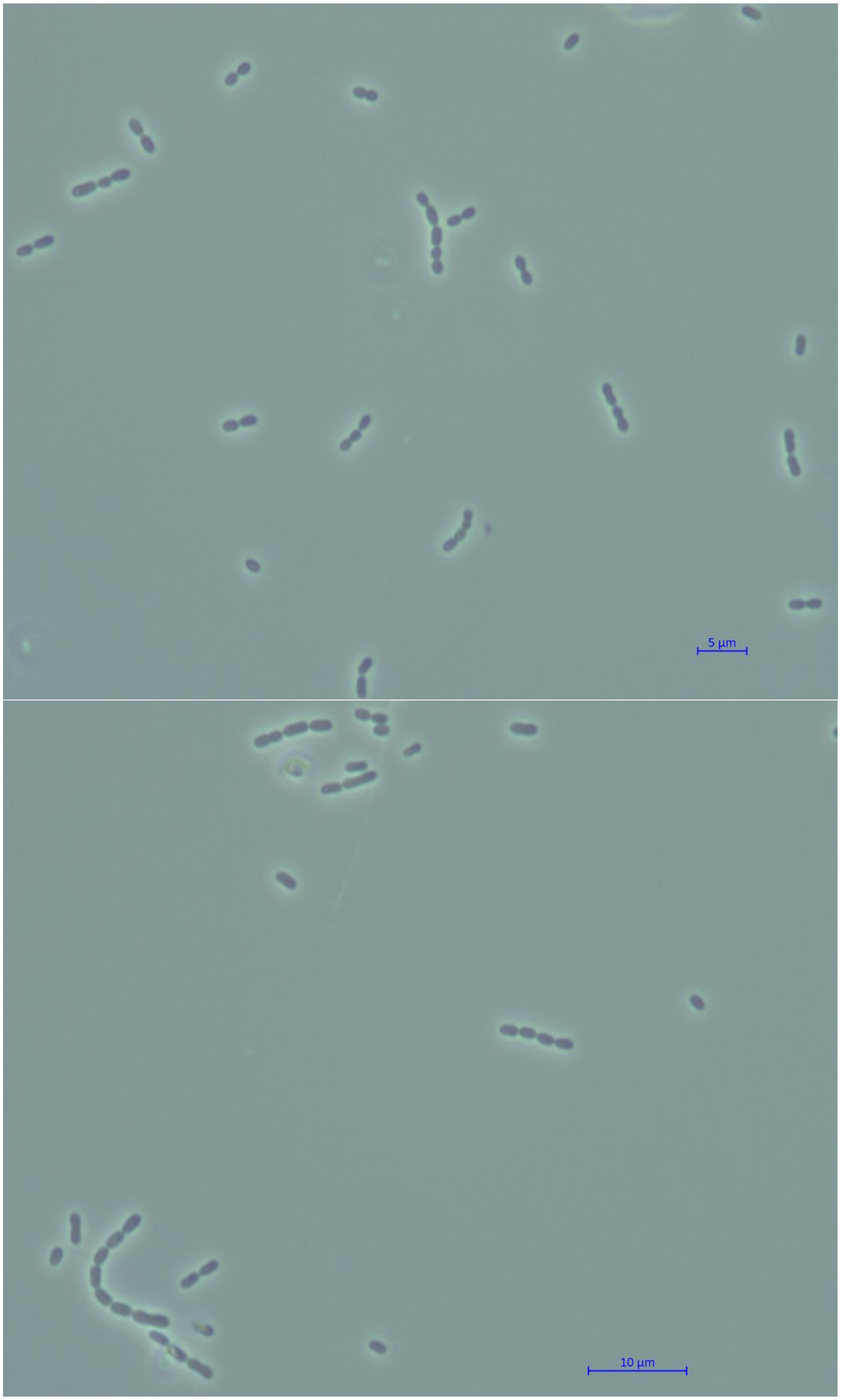

Figure S1. Cell morphology of strain AM2-LC observed by microscopy in 14-day-old culture.

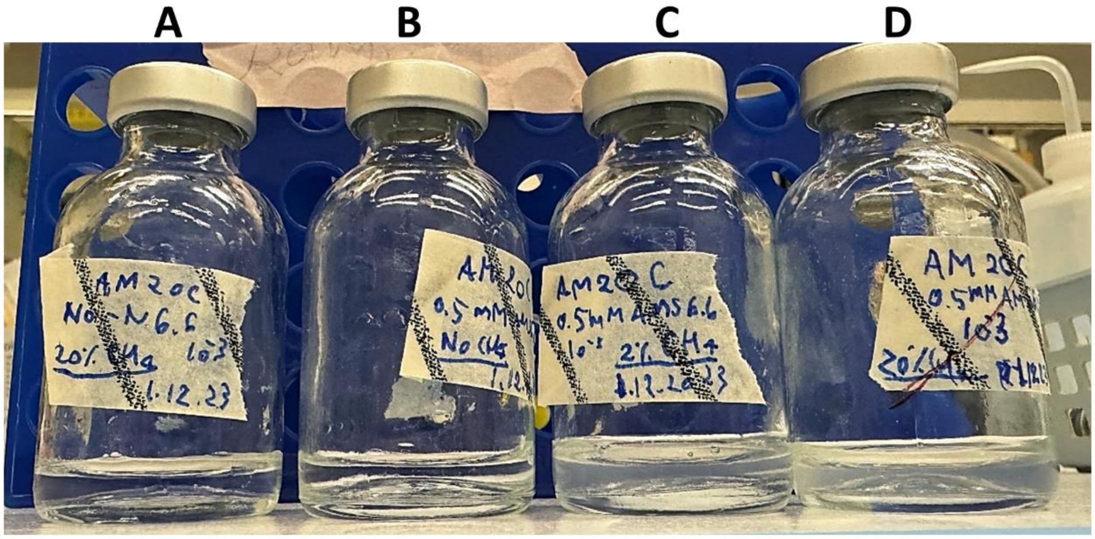

Figure S2. Growth of AM2-LC in the modified AMS medium, pH 6.6 after 6 days incubated statically at 20 °C (initial optical density (OD_{600nm}) on day 0 < 0.01) under four different conditions including (A) 80% air + 20% and CH_4 + AMS medium without ammonium addition (control), (B) 100% air and AMS medium without CH_4 (control), (C) 98% air + 2% CH_4 and AMS medium, and (D) 80% air + 20% and CH_4 + AMS medium.

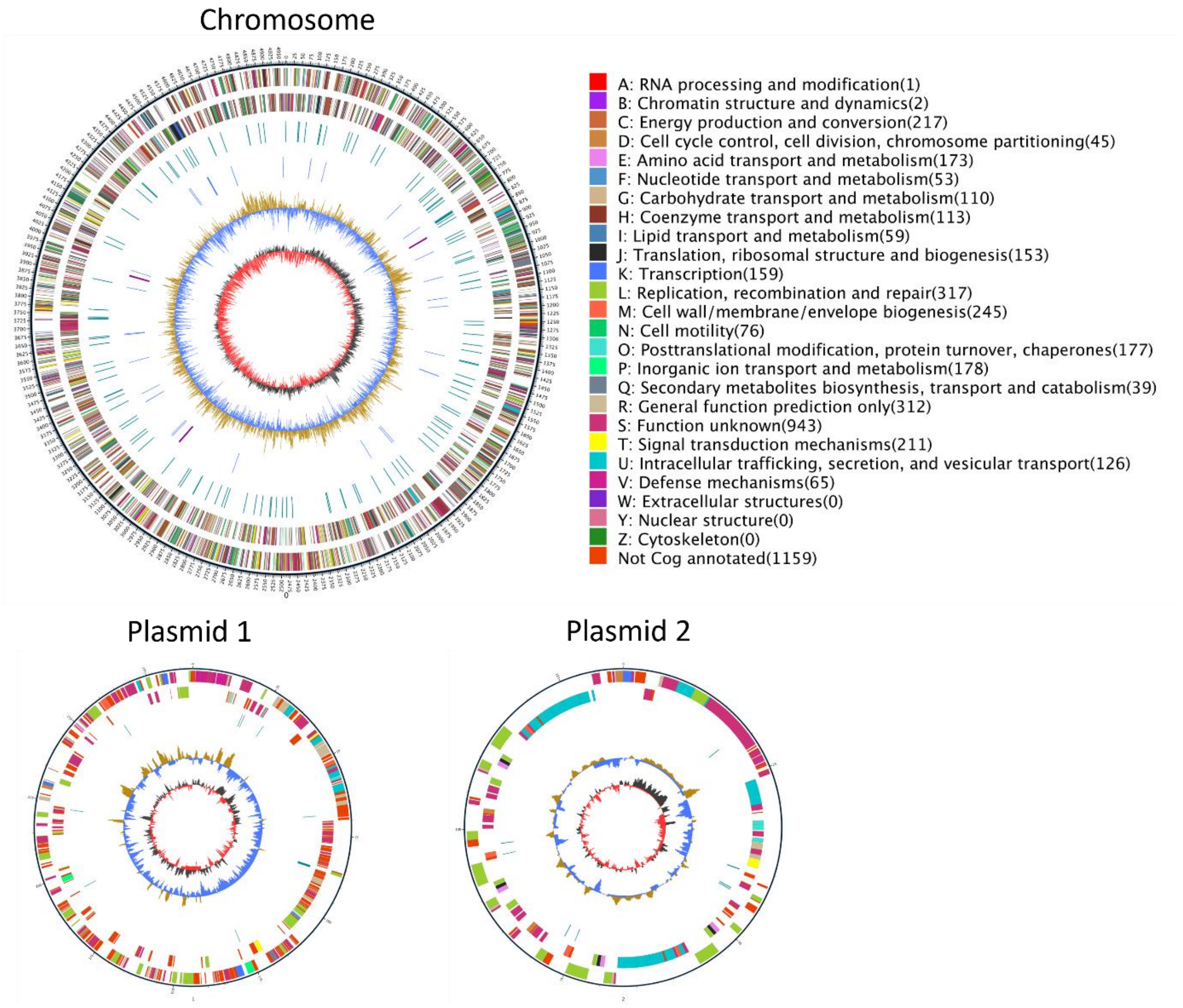

Figure S3. Circos diagrams of chromosome and plasmids of strain AM2-LC. The outer layer indicates the size of the chromosome/plasmid, in which each scale represents 5 kb in length. The second and third layer represents genes in + and – strand, respectively. The genes were colored according to COG classification. The fourth layer represents distribution of repetitive sequences. The fifth layer represents tRNA (blue) and rRNA (purple). The sixth layer indicates GC content. Peaks in light yellow indicates that corresponding GC content is higher than average level of entire genome. A higher peak indicates a larger difference compared with average GC content. Peaks in blue indicates corresponding GC content is lower than average. The inner layer represents GC-skew, where dark grey indicates region with G higher than C and red indicates the opposite.

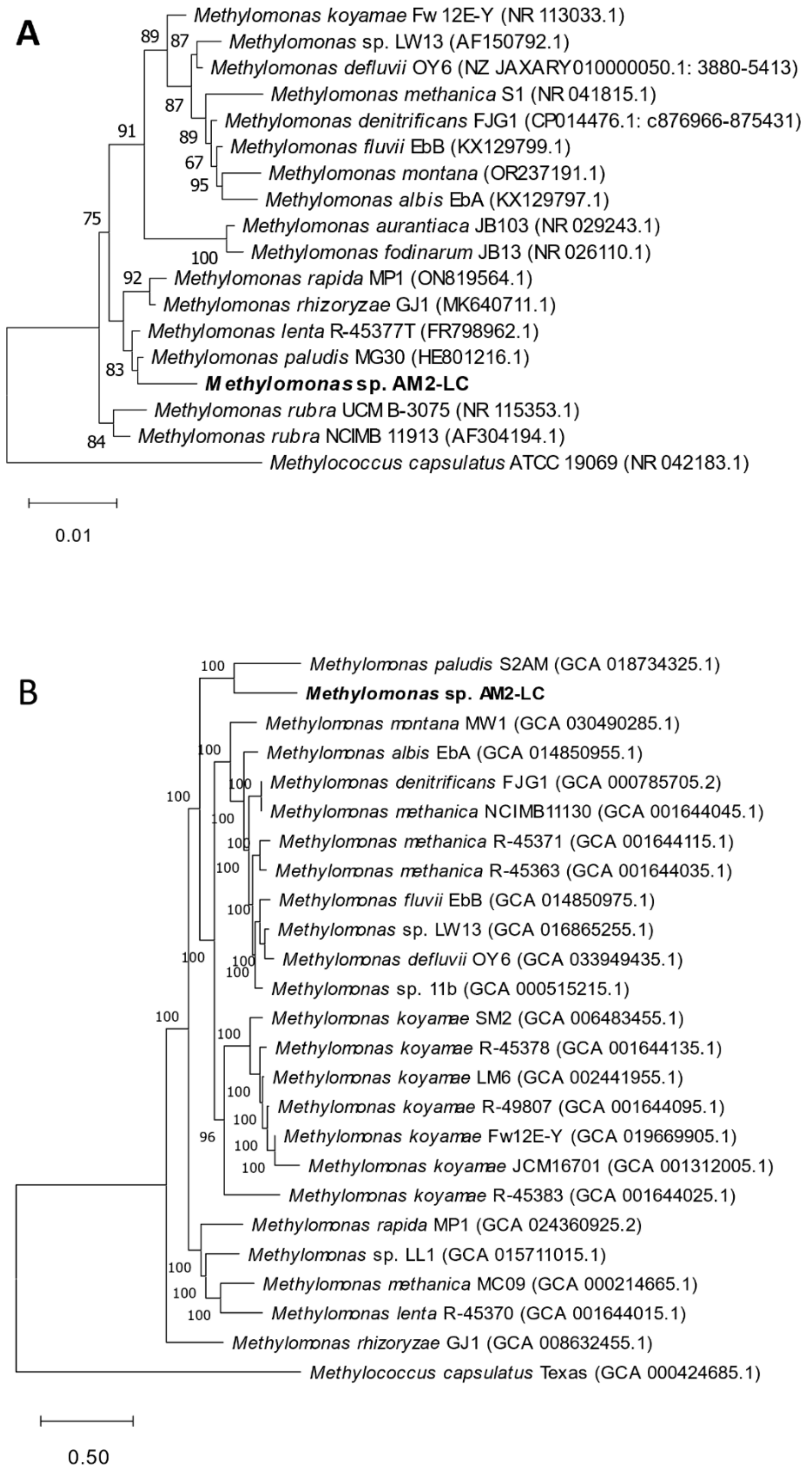

Figure S4. (A) Phylogenetic tree based on 16S rRNA genes and **(B)** Genome-wide phylogenomic tree (PhyloPhlAn). Both trees were constructed using the maximum likelihood algorithm with the GTR model for the 16S rRNA gene tree (in A) and PROTCATLG model for the genome-wide tree (in B). Numbers at the nodes indicate the percentage of occurrence in 100 bootstrapped trees. The scale bars indicate the number of substitutions per nucleotide (in A) and amino acid position (in B). Strain AM2-LC is highlighted in bold.
