# Supplementary material for: Genome of Methylomonas sp. AM2-LC, representing a methanotrophic bacterial species isolated from water column of a boreal, oxygen-stratified lake
Source: Front Genet. 2024 Aug 30;15:1440435. doi: 10.3389/fgene.2024.1440435 (PMC11392852; doi:10.3389/fgene.2024.1440435)
Supplement: Supplementary file 3 [file DataSheet2.ZIP › Supplementary_File_2/08.gene_cluster/AMSG_genome.zip!/index.html]

AMSG\_genome - 6 region(s) - antiSMASH results


antiSMASH version 6.0.1

Download

- Download all results
- Download GenBank summary file

About

Help

Contact

Select genomic region:

Overview

1.1

1.2

1.3

1.4

1.5

1.6

### Identified secondary metabolite regions using strictness 'relaxed'

**Contig00001**

| Region | Type | From | To | Most similar known cluster | | Similarity |
| --- | --- | --- | --- | --- | --- | --- |
| Region&nbsp1.1 | RiPP-like | 185,164 | 194,129 |  | | |
| Region&nbsp1.2 | T1PKS | 1,840,636 | 1,887,007 | myxochromide D | NRP:Cyclic depsipeptide + Polyketide:Iterative type I | 13% |
| Region&nbsp1.3 | RiPP-like | 2,972,847 | 2,983,689 |  | | |
| Region&nbsp1.4 | terpene | 3,458,439 | 3,472,301 |  | | |
| Region&nbsp1.5 | terpene | 4,490,249 | 4,513,308 | hopene | Terpene | 15% |
| Region&nbsp1.6 | redox-cofactor | 4,677,225 | 4,699,352 | lankacidin C | NRP + Polyketide | 13% |

| Region | Type | From | To | Most similar known cluster | | Similarity |
| --- | --- | --- | --- | --- | --- | --- |
| Region&nbsp1.1 | RiPP-like | 185,164 | 194,129 |  | | |
| Region&nbsp1.2 | T1PKS | 1,840,636 | 1,887,007 | myxochromide D | NRP:Cyclic depsipeptide + Polyketide:Iterative type I | 13% |
| Region&nbsp1.3 | RiPP-like | 2,972,847 | 2,983,689 |  | | |
| Region&nbsp1.4 | terpene | 3,458,439 | 3,472,301 |  | | |
| Region&nbsp1.5 | terpene | 4,490,249 | 4,513,308 | hopene | Terpene | 15% |
| Region&nbsp1.6 | redox-cofactor | 4,677,225 | 4,699,352 | lankacidin C | NRP + Polyketide | 13% |

No secondary metabolite regions were found in these records:
:   **Contig00002**
:   **Contig00003**

Compact view

Contig00001 - Region 1 - RiPP-like

Shows the layout of the region, marking coding sequences and areas of interest. Clicking a gene will select it and show any relevant details. Clicking an area feature (e.g. a candidate cluster) will select all coding sequences within that area. Double clicking an area feature will zoom to that area. Multiple genes and area features can be selected by clicking them while holding the Ctrl key.  
More detailed help is available here.

Download region GenBank file

Download region SVG

Location: 185,164 - 194,129 nt. (total: 8,966 nt)
Show pHMM detection rules used

RiPP-like: (strepbact or Antimicrobial14 or Bacteriocin\_IId or BacteriocIIc\_cy or Bacteriocin\_II or Bacteriocin\_IIi or Lactococcin or Antimicrobial17 or Lactococcin\_972 or Bacteriocin\_IIc or LcnG-beta or Cloacin or Linocin\_M18 or TIGR03651 or TIGR03693 or TIGR03601 or TIGR03795 or TIGR03975 or DUF692 or TIGR01193)

#### Legend:

core biosynthetic genes

additional biosynthetic genes

transport-related genes

regulatory genes

other genes

resistance

reset view

zoom to selection

Gene details

Shows details of the most recently selected gene, including names, products, location, and other annotations.

Select a gene to view the details available for it

ClusterBlast

KnownClusterBlast

SubClusterBlast

Similar gene clusters

Shows clusters from the antiSMASH database and other clusters of interest that are similar to the current region. Genes marked with the same colour are interrelated. White genes have no relationship.  
Click on reference genes to show details of similarities to genes within the current region.  
Click on an accession to open that entry in the antiSMASH database (if applicable).

All hits

NZ\_CP014360 (4571944-4582793): Methylomonas sp. DH-1, complete genome (53% of genes show similarity), bacteriocin

NZ\_CP023669 (4638784-4649633): Methylomonas koyamae strain LM6 chromosome, co... (53% of genes show similarity), bacteriocin

NZ\_KI911557 (2805542-2814278): Methylomonas sp. 11b scaffold1, whole genome s... (58% of genes show similarity), bacteriocin

NZ\_CP033381 (389867-398389): Methylomonas sp. LW13 chromosome, complete genome (53% of genes show similarity), bacteriocin

NC\_015572 (4869702-4880551): Methylomonas methanica MC09, complete sequence (50% of genes show similarity), bacteriocin

NZ\_CP043929 (4052-13742): Methylomonas sp. GJ1 chromosome, complete genome (50% of genes show similarity), bacteriocin

NZ\_JQKS01000019 (101704-112556): Methylobacter sp. BBA5.1 EK22DRAFT scaffold0... (40% of genes show similarity), bacteriocin

NZ\_KB912877 (1468426-1476650): Methylobacter marinus A45 MetmaDRAFT scaffold1... (44% of genes show similarity), bacteriocin

NZ\_PTIY01000007 (143619-154471): Methylobacter tundripaludum strain OWC-G53F ... (44% of genes show similarity), bacteriocin

NZ\_JH109152 (2018216-2029068): Methylobacter tundripaludum SV96 Mettuscaffold... (44% of genes show similarity), bacteriocin
Download graphic

Similar known gene clusters

Shows clusters from the MiBIG database that are similar to the current region. Genes marked with the same colour are interrelated. White genes have no relationship.  
Click on reference genes to show details of similarities to genes within the current region.  
Click on an accession to open that entry in the MiBIG database.

No matches found.

Similar subclusters

Shows sub-cluster units that are similar to the current region. Genes marked with the same colour are interrelated. White genes have no relationship.  
Click on reference genes to show details of similarities to genes within the current region.

No matches found.

Contig00001 - Region 2 - T1PKS

Shows the layout of the region, marking coding sequences and areas of interest. Clicking a gene will select it and show any relevant details. Clicking an area feature (e.g. a candidate cluster) will select all coding sequences within that area. Double clicking an area feature will zoom to that area. Multiple genes and area features can be selected by clicking them while holding the Ctrl key.  
More detailed help is available here.

Download region GenBank file

Download region SVG

Location: 1,840,636 - 1,887,007 nt. (total: 46,372 nt)
Show pHMM detection rules used

T1PKS: cds(PKS\_AT and (PKS\_KS or ene\_KS or mod\_KS or hyb\_KS or itr\_KS or tra\_KS))

#### Legend:

core biosynthetic genes

additional biosynthetic genes

transport-related genes

regulatory genes

other genes

resistance

reset view

zoom to selection

Gene details

Shows details of the most recently selected gene, including names, products, location, and other annotations.

Select a gene to view the details available for it

NRPS/PKS domains

ClusterBlast

KnownClusterBlast

SubClusterBlast

Detailed domain annotation

Shows NRPS- and PKS-related domains for each feature that contains them. Click on each domain for more information about the domain's location, consensus monomer prediction, and other details.  
A glossary is available here.

Selected features only

Show module domains

Similar gene clusters

Shows clusters from the antiSMASH database and other clusters of interest that are similar to the current region. Genes marked with the same colour are interrelated. White genes have no relationship.  
Click on reference genes to show details of similarities to genes within the current region.  
Click on an accession to open that entry in the antiSMASH database (if applicable).

All hits

NZ\_KE386569 (4149566-4241255): Methylobacter luteus IMV-B-3098 MetluDRAFT sca... (10% of genes show similarity), NRPS,T1PKS

NZ\_JAAOIX010000006 (23884-79260): Pseudomaricurvus alcaniphilus strain MEBiC0... (10% of genes show similarity), NRPS,T1PKS

NZ\_CP022684 (1746233-1789616): Ketobacter alkanivorans strain GI5 chromosome,... (10% of genes show similarity), T1PKS

NZ\_CP045118 (1296831-1353206): Pseudomonas sp. SCB32 chromosome, complete genome (8% of genes show similarity), T1PKS,bacteriocin

NZ\_FYEE01000002 (241729-287807): Pseudomonas sp. Irchel 3E20 isolate Pseudomo... (10% of genes show similarity), T1PKS

NZ\_JACHLI010000004 (32060-78153): Pseudomonas nitritireducens strain S00179 G... (12% of genes show similarity), T1PKS,bacteriocin

NZ\_WEIC01000003 (159877-205979): Amylibacter sp. SFDW26 contig3, whole genome... (8% of genes show similarity), T1PKS

NZ\_AP022347 (1019576-1067010): Rhodocyclaceae bacterium ICHIJ1 (8% of genes show similarity), T1PKS

NZ\_WWGV01000021 (12629-113444): Streptomyces sp. SID8356 SID8356.c21, whole g... (16% of genes show similarity), T1PKS,linaridin

NZ\_CP015362 (626362-773395): Streptomyces sp. S8 chromosome, complete genome (7% of genes show similarity), NRPS,T1PKS,linaridin,thioamide-NRP
Download graphic

Similar known gene clusters

Shows clusters from the MiBIG database that are similar to the current region. Genes marked with the same colour are interrelated. White genes have no relationship.  
Click on reference genes to show details of similarities to genes within the current region.  
Click on an accession to open that entry in the MiBIG database.

All hits

myxochromide D
Download graphic

Similar subclusters

Shows sub-cluster units that are similar to the current region. Genes marked with the same colour are interrelated. White genes have no relationship.  
Click on reference genes to show details of similarities to genes within the current region.

No matches found.

NRPS/PKS products

NRPS/PKS monomers

Predicted core structure(s)

Shows estimated product structure and polymer for each candidate cluster in the region. To show the product, click on the expander or the candidate cluster feature drawn in the overview.

For candidate cluster 2, location 1840635 - 1887007:

Rough prediction of core scaffold based on assumed PKS/NRPS colinearity; tailoring reactions not taken into account

**Polymer prediction:**
:   (redmal)

  
Direct lookup in NORINE database:
strict
or
relaxed

Link to NORINE database query form

NRPS/PKS monomer predictions

Shows the predicted monomers for each adynelation domain and acyltransferase within genes. Each gene prediction can be expanded to view detailed predictions of each domain. Each prediction can be expanded to view the predictions by tool (and, for some tools, further expanded for extra details).

**GE001685**: mal

:   **PKS\_AT (553..848)**: mal

    ATSignature: Malonyl-CoA

    Top 3 matches:
    :   Malonyl-CoA: 83.3%
    :   CE-Malonyl-CoA: 62.5%
    :   Acetyl-CoA: 58.3%

      
    minowa: Malonyl-CoA

    Prediction, score:
    :   Malonyl-CoA: 105.8


        Methoxymalonyl-CoA: 92.8


        Methylmalonyl-CoA: 91.5


        Isobutyryl-CoA: 71.6


        Ethylmalonyl-CoA: 57.2


        inactive: 47.4


        Propionyl-CoA: 44.4


        Acetyl-CoA: 40.7


        trans-1,2-CPDA: 38.0


        2-Methylbutyryl-CoA: 36.0


        fatty\_acid: 35.6


        Benzoyl-CoA: 29.7


        3-Methylbutyryl-CoA: 24.8


        CHC-CoA: 11.6

Contig00001 - Region 3 - RiPP-like

Shows the layout of the region, marking coding sequences and areas of interest. Clicking a gene will select it and show any relevant details. Clicking an area feature (e.g. a candidate cluster) will select all coding sequences within that area. Double clicking an area feature will zoom to that area. Multiple genes and area features can be selected by clicking them while holding the Ctrl key.  
More detailed help is available here.

Download region GenBank file

Download region SVG

Location: 2,972,847 - 2,983,689 nt. (total: 10,843 nt)
Show pHMM detection rules used

RiPP-like: (strepbact or Antimicrobial14 or Bacteriocin\_IId or BacteriocIIc\_cy or Bacteriocin\_II or Bacteriocin\_IIi or Lactococcin or Antimicrobial17 or Lactococcin\_972 or Bacteriocin\_IIc or LcnG-beta or Cloacin or Linocin\_M18 or TIGR03651 or TIGR03693 or TIGR03601 or TIGR03795 or TIGR03975 or DUF692 or TIGR01193)

#### Legend:

core biosynthetic genes

additional biosynthetic genes

transport-related genes

regulatory genes

other genes

resistance

reset view

zoom to selection

Gene details

Shows details of the most recently selected gene, including names, products, location, and other annotations.

Select a gene to view the details available for it

ClusterBlast

KnownClusterBlast

SubClusterBlast

Similar gene clusters

Shows clusters from the antiSMASH database and other clusters of interest that are similar to the current region. Genes marked with the same colour are interrelated. White genes have no relationship.  
Click on reference genes to show details of similarities to genes within the current region.  
Click on an accession to open that entry in the antiSMASH database (if applicable).

All hits

NZ\_JQKS01000046 (15236-26109): Methylobacter sp. BBA5.1 EK22DRAFT scaffold000... (23% of genes show similarity), bacteriocin

NZ\_KB912877 (3338863-3349736): Methylobacter marinus A45 MetmaDRAFT scaffold1... (22% of genes show similarity), bacteriocin

NZ\_CP044205 (1056209-1083778): Candidatus Methylospira mobilis strain Shm1 ch... (11% of genes show similarity), bacteriocin

NC\_015572 (4719996-4729370): Methylomonas methanica MC09, complete sequence (27% of genes show similarity), bacteriocin

NZ\_CP033381 (2847939-2858812): Methylomonas sp. LW13 chromosome, complete genome (25% of genes show similarity), bacteriocin

NZ\_CP024202 (2738657-2749506): Methylomicrobium sp. wino1 chromosome, complet... (27% of genes show similarity), bacteriocin

NZ\_QGDM01000018 (124293-135139): Pseudomonas sp. 43mfcvi1.1 Ga0073240 118, wh... (33% of genes show similarity), bacteriocin

NC\_002516 (4584490-4595321): Pseudomonas aeruginosa PAO1, complete genome (27% of genes show similarity), bacteriocin

NC\_011770 (940030-950721): Pseudomonas aeruginosa LESB58, complete genome (25% of genes show similarity), bacteriocin

NZ\_KI518856 (73187-83877): Pseudomonas aeruginosa BL12 adgfc-supercont1.1, wh... (25% of genes show similarity), bacteriocin
Download graphic

Similar known gene clusters

Shows clusters from the MiBIG database that are similar to the current region. Genes marked with the same colour are interrelated. White genes have no relationship.  
Click on reference genes to show details of similarities to genes within the current region.  
Click on an accession to open that entry in the MiBIG database.

No matches found.

Similar subclusters

Shows sub-cluster units that are similar to the current region. Genes marked with the same colour are interrelated. White genes have no relationship.  
Click on reference genes to show details of similarities to genes within the current region.

No matches found.

Contig00001 - Region 4 - terpene

Shows the layout of the region, marking coding sequences and areas of interest. Clicking a gene will select it and show any relevant details. Clicking an area feature (e.g. a candidate cluster) will select all coding sequences within that area. Double clicking an area feature will zoom to that area. Multiple genes and area features can be selected by clicking them while holding the Ctrl key.  
More detailed help is available here.

Download region GenBank file

Download region SVG

Location: 3,458,439 - 3,472,301 nt. (total: 13,863 nt)
Show pHMM detection rules used

terpene: (Terpene\_synth or Terpene\_synth\_C or phytoene\_synt or Lycopene\_cycl or terpene\_cyclase or NapT7 or fung\_ggpps or fung\_ggpps2 or trichodiene\_synth or TRI5)

#### Legend:

core biosynthetic genes

additional biosynthetic genes

transport-related genes

regulatory genes

other genes

resistance

reset view

zoom to selection

Gene details

Shows details of the most recently selected gene, including names, products, location, and other annotations.

Select a gene to view the details available for it

ClusterBlast

KnownClusterBlast

SubClusterBlast

Similar gene clusters

Shows clusters from the antiSMASH database and other clusters of interest that are similar to the current region. Genes marked with the same colour are interrelated. White genes have no relationship.  
Click on reference genes to show details of similarities to genes within the current region.  
Click on an accession to open that entry in the antiSMASH database (if applicable).

All hits

NZ\_JH109152 (600884-619617): Methylobacter tundripaludum SV96 Mettuscaffold 1... (43% of genes show similarity), terpene

NZ\_KI911557 (1716224-1735303): Methylomonas sp. 11b scaffold1, whole genome s... (47% of genes show similarity), terpene

NZ\_CP024202 (4037231-4058014): Methylomicrobium sp. wino1 chromosome, complet... (42% of genes show similarity), terpene

NZ\_KB913025 (2931829-2953794): Methylovulum miyakonense HT12 MetmiDRAFT scaff... (17% of genes show similarity), terpene

NZ\_CP035467 (3236774-3254501): Methylomicrobium buryatense strain 5GB1C chrom... (27% of genes show similarity), terpene

NC\_016112 (2893693-2911894): Methylomicrobium alcaliphilum 20Z, complete genome (26% of genes show similarity), terpene

NZ\_JACHIE010000010 (48854-70954): Acetobacter lovaniensis strain DSM 4491 Ga0... (16% of genes show similarity), terpene

NZ\_CP043929 (1957957-1979592): Methylomonas sp. GJ1 chromosome, complete genome (23% of genes show similarity), terpene

NZ\_CP023669 (2080018-2101986): Methylomonas koyamae strain LM6 chromosome, co... (14% of genes show similarity), terpene

NC\_015572 (2700526-2721126): Methylomonas methanica MC09, complete sequence (13% of genes show similarity), terpene
Download graphic

Similar known gene clusters

Shows clusters from the MiBIG database that are similar to the current region. Genes marked with the same colour are interrelated. White genes have no relationship.  
Click on reference genes to show details of similarities to genes within the current region.  
Click on an accession to open that entry in the MiBIG database.

No matches found.

Similar subclusters

Shows sub-cluster units that are similar to the current region. Genes marked with the same colour are interrelated. White genes have no relationship.  
Click on reference genes to show details of similarities to genes within the current region.

No matches found.

Contig00001 - Region 5 - terpene

Shows the layout of the region, marking coding sequences and areas of interest. Clicking a gene will select it and show any relevant details. Clicking an area feature (e.g. a candidate cluster) will select all coding sequences within that area. Double clicking an area feature will zoom to that area. Multiple genes and area features can be selected by clicking them while holding the Ctrl key.  
More detailed help is available here.

Download region GenBank file

Download region SVG

Location: 4,490,249 - 4,513,308 nt. (total: 23,060 nt)
Show pHMM detection rules used

terpene: (Terpene\_synth or Terpene\_synth\_C or phytoene\_synt or Lycopene\_cycl or terpene\_cyclase or NapT7 or fung\_ggpps or fung\_ggpps2 or trichodiene\_synth or TRI5)

#### Legend:

core biosynthetic genes

additional biosynthetic genes

transport-related genes

regulatory genes

other genes

resistance

reset view

zoom to selection

Gene details

Shows details of the most recently selected gene, including names, products, location, and other annotations.

Select a gene to view the details available for it

ClusterBlast

KnownClusterBlast

SubClusterBlast

Similar gene clusters

Shows clusters from the antiSMASH database and other clusters of interest that are similar to the current region. Genes marked with the same colour are interrelated. White genes have no relationship.  
Click on reference genes to show details of similarities to genes within the current region.  
Click on an accession to open that entry in the antiSMASH database (if applicable).

All hits

NZ\_CP033381 (1111784-1134841): Methylomonas sp. LW13 chromosome, complete genome (73% of genes show similarity), terpene

NZ\_KI911557 (2179227-2202287): Methylomonas sp. 11b scaffold1, whole genome s... (70% of genes show similarity), terpene

NC\_015572 (3825954-3849017): Methylomonas methanica MC09, complete sequence (60% of genes show similarity), terpene

NZ\_CP023669 (1331466-1353533): Methylomonas koyamae strain LM6 chromosome, co... (73% of genes show similarity), terpene

NZ\_CP014360 (1297116-1320176): Methylomonas sp. DH-1, complete genome (77% of genes show similarity), terpene

NZ\_CP043929 (4247178-4270238): Methylomonas sp. GJ1 chromosome, complete genome (66% of genes show similarity), terpene

NZ\_KB889965 (1586394-1607654): Methylosarcina fibrata AML-C10 A3OWDRAFT scaff... (77% of genes show similarity), terpene

NZ\_CM001475 (3218860-3241926): Methylomicrobium album BG8 chromosome, whole g... (70% of genes show similarity), terpene

NZ\_KB912877 (4148160-4171228): Methylobacter marinus A45 MetmaDRAFT scaffold1... (73% of genes show similarity), terpene

NZ\_KE386569 (1742213-1763167): Methylobacter luteus IMV-B-3098 MetluDRAFT sca... (63% of genes show similarity), terpene
Download graphic

Similar known gene clusters

Shows clusters from the MiBIG database that are similar to the current region. Genes marked with the same colour are interrelated. White genes have no relationship.  
Click on reference genes to show details of similarities to genes within the current region.  
Click on an accession to open that entry in the MiBIG database.

All hits

hopene
Download graphic

Similar subclusters

Shows sub-cluster units that are similar to the current region. Genes marked with the same colour are interrelated. White genes have no relationship.  
Click on reference genes to show details of similarities to genes within the current region.

No matches found.

Contig00001 - Region 6 - redox-cofactor

Shows the layout of the region, marking coding sequences and areas of interest. Clicking a gene will select it and show any relevant details. Clicking an area feature (e.g. a candidate cluster) will select all coding sequences within that area. Double clicking an area feature will zoom to that area. Multiple genes and area features can be selected by clicking them while holding the Ctrl key.  
More detailed help is available here.

Download region GenBank file

Download region SVG

Location: 4,677,225 - 4,699,352 nt. (total: 22,128 nt)
Show pHMM detection rules used

redox-cofactor: (cds(PF04055 and (TIGR04085 or SPASM)) and (TIGR02111 or TIGR03966) and minimum(1, [Bottromycin\_Methyltransferase\_RRE, Cyanobactin\_D\_RRE, Cytolysin\_C\_RRE, Goadsporin\_CD\_RRE, Heterocycloanthracin\_C\_RRE, Lanthipeptide\_LanB\_RRE, Lasso\_Fused\_RRE, Mycofactocin\_RRE, NHLP\_CD\_RRE, Other\_Sactipeptide\_rSAM\_RRE, Pantocin\_Microcin\_RRE, Plantazolicin\_C\_RRE, PlpY\_RRE, PqqD\_RRE, Proteusin\_Epimerase\_RRE, Proteusin\_Methyltransferase\_RRE, Quinohemoprotein\_rSAM\_RRE, Ranthipeptide\_rSAM\_RRE, Stand\_Alone\_Lasso\_RRE, Streptide\_RRE, Subtilosin\_rSAM\_RRE, Thiaglutamate\_B\_RRE, Thiaglutamate\_G\_RRE, Thiaglutamate\_I\_RRE, Thiopeptide\_F\_RRE, Thuricin\_rSAM\_RRE, Thurincin\_rSAM\_RRE, Trifolitoxin\_RRE]))

#### Legend:

core biosynthetic genes

additional biosynthetic genes

transport-related genes

regulatory genes

other genes

resistance

reset view

zoom to selection

Gene details

Shows details of the most recently selected gene, including names, products, location, and other annotations.

Select a gene to view the details available for it

ClusterBlast

KnownClusterBlast

SubClusterBlast

Similar gene clusters

Shows clusters from the antiSMASH database and other clusters of interest that are similar to the current region. Genes marked with the same colour are interrelated. White genes have no relationship.  
Click on reference genes to show details of similarities to genes within the current region.  
Click on an accession to open that entry in the antiSMASH database (if applicable).

All hits

NZ\_CP005970 (3160121-3201723): Pseudomonas syringae UMAF0158 chromosome, comp... (14% of genes show similarity), NRPS-like

NZ\_CP007014 (516132-557785): Pseudomonas syringae CC1557, complete sequence (14% of genes show similarity), NRPS-like

NZ\_CP013183 (5322605-5365481): Pseudomonas syringae pv. lapsa strain ATCC 108... (15% of genes show similarity), NRPS-like

NZ\_JACHCU010000005 (375513-414865): Herbaspirillum sp. SJZ102 Ga0401611 05, w... (12% of genes show similarity), thiopeptide

NC\_010682 (299562-340780): Ralstonia pickettii 12J chromosome 1, complete seq... (10% of genes show similarity), arylpolyene

NZ\_JH815236 (1357854-1399072): Ralstonia sp. 5 2 56FAA supercont2.1, whole ge... (10% of genes show similarity), arylpolyene

NZ\_LT963391 (4458122-4502163): Pseudomonas syringae pv. cerasicola isolate CF... (10% of genes show similarity), NRPS-like

NZ\_CM001763 (1417411-1458684): Pseudomonas syringae pv. syringae B64 chromoso... (10% of genes show similarity), NRPS-like

NZ\_CM001986 (5503448-5544229): Pseudomonas syringae pv. syringae SM chromosom... (10% of genes show similarity), NRPS-like

NZ\_KB316543 (28005-72046): Pseudomonas syringae BRIP34881 scaffold13, whole g... (9% of genes show similarity), NRPS-like
Download graphic

Similar known gene clusters

Shows clusters from the MiBIG database that are similar to the current region. Genes marked with the same colour are interrelated. White genes have no relationship.  
Click on reference genes to show details of similarities to genes within the current region.  
Click on an accession to open that entry in the MiBIG database.

All hits

lankacidin C
Download graphic

Similar subclusters

Shows sub-cluster units that are similar to the current region. Genes marked with the same colour are interrelated. White genes have no relationship.  
Click on reference genes to show details of similarities to genes within the current region.

No matches found.

If you have found antiSMASH useful, please cite us.
