# Supplementary material for: Genome of Methylomonas sp. AM2-LC, representing a methanotrophic bacterial species isolated from water column of a boreal, oxygen-stratified lake
Source: Front Genet. 2024 Aug 30;15:1440435. doi: 10.3389/fgene.2024.1440435 (PMC11392852; doi:10.3389/fgene.2024.1440435)
Supplement: Supplementary file 3 [file DataSheet2.ZIP › Supplementary_File_2/08.gene_cluster/AMSG_genome.zip!/knownclusterblast/region2/GE001668_mibig_hits.html]

| MIBiG Protein | Description | MIBiG Cluster | MiBiG Product | % ID | % Coverage | BLAST Score | E-value |
| --- | --- | --- | --- | --- | --- | --- | --- |
| AFB74478.1 | putative\_response\_regulator | BGC0000550 | RiPP:Lanthipeptide | 36.0 | 45.3 | 62.0 | 8.9e-10 |
| ACX68642.1 | SivR | BGC0000547 | RiPP:Lanthipeptide | 37.0 | 44.2 | 62.0 | 1.5e-09 |
| ABI30224.1 | McdR | BGC0000526 | RiPP:Lanthipeptide | 34.0 | 45.3 | 61.0 | 2.6e-09 |
| AOT85897.1 | response\_regulator | BGC0001788 | RiPP | 35.0 | 45.3 | 61.0 | 2.6e-09 |
| AAB92599.1 | ScnR | BGC0000557 | RiPP:Lanthipeptide | 34.0 | 45.3 | 58.0 | 1.7e-08 |
| AEG64693.1 | two\_component\_system\_response\_regulator | BGC0000379 | NRP | 29.0 | 43.4 | 56.0 | 6.4e-08 |
| ADM69285.1 | transcriptional\_response\_regulator | BGC0001727 | RiPP | 28.0 | 43.4 | 52.0 | 1.2e-06 |
