# Supplementary material for: Genome of Methylomonas sp. AM2-LC, representing a methanotrophic bacterial species isolated from water column of a boreal, oxygen-stratified lake
Source: Front Genet. 2024 Aug 30;15:1440435. doi: 10.3389/fgene.2024.1440435 (PMC11392852; doi:10.3389/fgene.2024.1440435)
Supplement: Supplementary file 3 [file DataSheet2.ZIP › Supplementary_File_2/08.gene_cluster/AMSG_genome.zip!/knownclusterblast/region2/GE001681_mibig_hits.html]

| MIBiG Protein | Description | MIBiG Cluster | MiBiG Product | % ID | % Coverage | BLAST Score | E-value |
| --- | --- | --- | --- | --- | --- | --- | --- |
| YP\_002539319.1 | transposase\_InsI\_for\_insertion\_sequence\_element\_IS30B/C/D | BGC0001555 | RiPP | 48.0 | 95.9 | 350.0 | 3.1e-96 |
| ADX66471.1 | Tnp | BGC0000108 | Polyketide | 43.0 | 96.6 | 269.0 | 6.9e-72 |
