# Supplementary material for: Genome of Methylomonas sp. AM2-LC, representing a methanotrophic bacterial species isolated from water column of a boreal, oxygen-stratified lake
Source: Front Genet. 2024 Aug 30;15:1440435. doi: 10.3389/fgene.2024.1440435 (PMC11392852; doi:10.3389/fgene.2024.1440435)
Supplement: Supplementary file 3 [file DataSheet2.ZIP › Supplementary_File_2/08.gene_cluster/AMSG_genome.zip!/knownclusterblast/region2/GE001671_mibig_hits.html]

| MIBiG Protein | Description | MIBiG Cluster | MiBiG Product | % ID | % Coverage | BLAST Score | E-value |
| --- | --- | --- | --- | --- | --- | --- | --- |
| AMK92553.1 | 5-methyltetrahydropteroyltriglutamate--\_homocysteine\_methyltransferase | BGC0001815 | Polyketide | 62.0 | 100.0 | 428.0 | 1.1e-119 |
| AAL06650.1 | truncated\_methionine\_synthase-like\_protein | BGC0000965 | Polyketide:Iterative type I + Polyketide:Enediyne type I | 59.0 | 100.0 | 423.0 | 4.5e-118 |
