# Supplementary material for: Genome of Methylomonas sp. AM2-LC, representing a methanotrophic bacterial species isolated from water column of a boreal, oxygen-stratified lake
Source: Front Genet. 2024 Aug 30;15:1440435. doi: 10.3389/fgene.2024.1440435 (PMC11392852; doi:10.3389/fgene.2024.1440435)
Supplement: Supplementary file 3 [file DataSheet2.ZIP › Supplementary_File_2/08.gene_cluster/AMSG_genome.zip!/knownclusterblast/region2/GE001664_mibig_hits.html]

| MIBiG Protein | Description | MIBiG Cluster | MiBiG Product | % ID | % Coverage | BLAST Score | E-value |
| --- | --- | --- | --- | --- | --- | --- | --- |
| AQZ37121.1 | RNA\_polymerase\_sigma\_factor | BGC0001511 | Polyketide | 35.0 | 96.0 | 179.0 | 8e-45 |
| AAM54109.1 | RNA\_polymerase\_sigma\_factor | BGC0000020 | Polyketide | 34.0 | 96.0 | 178.0 | 2.3e-44 |
| BAF98617.1 | putative\_DNA\_helicase | BGC0000651 | Terpene | 32.0 | 72.6 | 106.0 | 1.1e-22 |
| AGU42435.1 | Orf27 | BGC0000842 | Other:Non-NRP beta-lactam | 30.0 | 70.5 | 78.0 | 2.5e-14 |
| WP\_018891761.1 | SigB/SigF/SigG\_family\_RNA\_polymerase\_sigma\_factor | BGC0001558 | Polyketide | 25.0 | 71.4 | 71.0 | 4e-12 |
| ABS90485.1 | RNA\_polymerase\_sigma\_factor | BGC0001106 | NRP + Polyketide | 25.0 | 66.6 | 58.0 | 2.7e-08 |
| BAE95539.1 | putative\_RNA\_polymerase\_sigma\_factor | BGC0000703 | Saccharide | 22.0 | 70.5 | 54.0 | 3e-07 |
