# Supplementary material for: Genome of Methylomonas sp. AM2-LC, representing a methanotrophic bacterial species isolated from water column of a boreal, oxygen-stratified lake
Source: Front Genet. 2024 Aug 30;15:1440435. doi: 10.3389/fgene.2024.1440435 (PMC11392852; doi:10.3389/fgene.2024.1440435)
Supplement: Supplementary file 3 [file DataSheet2.ZIP › Supplementary_File_2/08.gene_cluster/AMSG_genome.zip!/knownclusterblast/region2/GE001683_mibig_hits.html]

| MIBiG Protein | Description | MIBiG Cluster | MiBiG Product | % ID | % Coverage | BLAST Score | E-value |
| --- | --- | --- | --- | --- | --- | --- | --- |
| XP\_001827205.1 |  | BGC0001996 | Other | 33.0 | 90.7 | 222.0 | 1.1e-57 |
| AMY15071.1 | citrate\_synthase | BGC0001339 | Polyketide:Iterative type I | 33.0 | 91.0 | 180.0 | 8e-45 |
