# Supplementary material for: Genome of Methylomonas sp. AM2-LC, representing a methanotrophic bacterial species isolated from water column of a boreal, oxygen-stratified lake
Source: Front Genet. 2024 Aug 30;15:1440435. doi: 10.3389/fgene.2024.1440435 (PMC11392852; doi:10.3389/fgene.2024.1440435)
Supplement: Supplementary file 3 [file DataSheet2.ZIP › Supplementary_File_2/08.gene_cluster/AMSG_genome.zip!/knownclusterblast/region2/GE001686_mibig_hits.html]

| MIBiG Protein | Description | MIBiG Cluster | MiBiG Product | % ID | % Coverage | BLAST Score | E-value |
| --- | --- | --- | --- | --- | --- | --- | --- |
| orf6 | 8-amino-7-oxononanoate\_synthase | BGC0001420 | NRP:Cyclic depsipeptide + Polyketide:Iterative type I | 47.0 | 79.8 | 323.0 | 6e-88 |
| APZ78719.1 | 8-amino-7-oxononanoate\_synthase | BGC0001420 | NRP:Cyclic depsipeptide + Polyketide:Iterative type I | 47.0 | 79.8 | 323.0 | 6e-88 |
| BAG84248.1 | putative\_polyketide\_synthase | BGC0000257 | Polyketide | 42.0 | 86.6 | 308.0 | 1.5e-83 |
| CAA16183.1 | polyketide\_synthase | BGC0001063 | NRP + Polyketide | 45.0 | 80.2 | 303.0 | 6.4e-82 |
| AHF22854.1 | MarL | BGC0000091 | Polyketide | 42.0 | 86.4 | 300.0 | 3.2e-81 |
| CAA16181.1 | putative\_8-amino-7-oxononanoate\_synthase | BGC0001063 | NRP + Polyketide | 31.0 | 77.0 | 193.0 | 7.2e-49 |
| AHF22852.1 | MarN | BGC0000091 | Polyketide | 30.0 | 76.4 | 192.0 | 1.2e-48 |
| BAG84263.1 | putative\_4-hydroxy-2,2'-bipyrrole-4-methanol\_synthase | BGC0000257 | Polyketide | 29.0 | 78.6 | 188.0 | 2.3e-47 |
| CRI73789.1 | glycine\_acetyltransferase | BGC0001215 | NRP | 33.0 | 78.0 | 182.0 | 1.3e-45 |
| ABB69080.1 | putative\_HBM\_synthase/aminotransferase | BGC0000260 | Polyketide | 28.0 | 79.3 | 179.0 | 1.4e-44 |
| ABJ90148.1 | MoeC4 | BGC0000805 | Saccharide | 31.0 | 86.8 | 161.0 | 3.9e-39 |
| ACB12555.1 | Fum8 | BGC0000063 | Polyketide | 23.0 | 70.7 | 67.0 | 5.9e-11 |
