# Supplementary material for: Genome of Methylomonas sp. AM2-LC, representing a methanotrophic bacterial species isolated from water column of a boreal, oxygen-stratified lake
Source: Front Genet. 2024 Aug 30;15:1440435. doi: 10.3389/fgene.2024.1440435 (PMC11392852; doi:10.3389/fgene.2024.1440435)
Supplement: Supplementary file 3 [file DataSheet2.ZIP › Supplementary_File_2/08.gene_cluster/AMSG_genome.zip!/knownclusterblast/region2/GE001687_mibig_hits.html]

| MIBiG Protein | Description | MIBiG Cluster | MiBiG Product | % ID | % Coverage | BLAST Score | E-value |
| --- | --- | --- | --- | --- | --- | --- | --- |
| AEH59111.1 | exporters\_of\_the\_RND\_superfamily-like\_protein | BGC0000385 | NRP | 27.0 | 73.7 | 156.0 | 1.3e-37 |
