# Supplementary material for: Genome of Methylomonas sp. AM2-LC, representing a methanotrophic bacterial species isolated from water column of a boreal, oxygen-stratified lake
Source: Front Genet. 2024 Aug 30;15:1440435. doi: 10.3389/fgene.2024.1440435 (PMC11392852; doi:10.3389/fgene.2024.1440435)
Supplement: Supplementary file 3 [file DataSheet2.ZIP › Supplementary_File_2/08.gene_cluster/AMSG_genome.zip!/knownclusterblast/region5/GE004007_mibig_hits.html]

| MIBiG Protein | Description | MIBiG Cluster | MiBiG Product | % ID | % Coverage | BLAST Score | E-value |
| --- | --- | --- | --- | --- | --- | --- | --- |
| AEE75535.1 | Terpenoid\_synthases\_superfamily\_protein | BGC0001756 | Terpene | 46.0 | 100.7 | 220.0 | 2.8e-57 |
| ATN39904.1 | MstJ | BGC0001664 | Terpene | 45.0 | 99.3 | 220.0 | 4.8e-57 |
| AEE75537.1 | geranylgeranyl\_pyrophosphate\_synthase\_3 | BGC0001756 | Terpene | 46.0 | 100.7 | 219.0 | 8.2e-57 |
| ANM64719.1 | geranylgeranyl\_pyrophosphate\_synthase\_3 | BGC0001756 | Terpene | 46.0 | 100.7 | 219.0 | 8.2e-57 |
| ANM64718.1 | geranylgeranyl\_pyrophosphate\_synthase\_3 | BGC0001756 | Terpene | 46.0 | 100.7 | 219.0 | 8.2e-57 |
| NP\_443950.1 | geranyltranstransferase | BGC0001605 | Terpene | 46.0 | 93.9 | 217.0 | 4.1e-56 |
| AHI58825.1 | geranyl\_diphosphate\_synthase | BGC0000668 | Terpene + Alkaloid | 44.0 | 99.7 | 211.0 | 2.2e-54 |
| APZ79611.1 | FisE2 | BGC0001595 | Alkaloid | 44.0 | 99.3 | 210.0 | 3.8e-54 |
| AEE75533.1 | Polyprenyl\_synthetase\_family\_protein | BGC0001756 | Terpene | 46.0 | 91.6 | 206.0 | 5.5e-53 |
| AHB62766.1 | geranyl\_diphosphate\_synthase | BGC0001126 | Terpene + Alkaloid | 42.0 | 99.7 | 202.0 | 8e-52 |
| APZ79589.1 | FilE2 | BGC0001501 | Alkaloid | 42.0 | 99.7 | 202.0 | 8e-52 |
| APZ79556.1 | FimE2 | BGC0001594 | Alkaloid | 42.0 | 99.7 | 202.0 | 8e-52 |
| APB62252.1 | polyprenyl\_synthetase | BGC0001612 | Alkaloid | 42.0 | 99.7 | 202.0 | 8e-52 |
| AAZ73127.1 | geranylgeranyl\_pyrophosphate\_synthase | BGC0000639 | Terpene | 40.0 | 88.6 | 148.0 | 2.3e-35 |
| AAZ73139.1 | geranylgeranyl\_pyrophosphate\_synthase | BGC0000641 | Terpene | 39.0 | 87.5 | 139.0 | 8.3e-33 |
| AAZ73145.1 | geranylgeranyl\_pyrophosphate\_synthase | BGC0000642 | Terpene | 38.0 | 87.2 | 135.0 | 1.6e-31 |
| AAZ73133.1 | geranylgeranyl\_pyrophosphate\_synthase | BGC0000640 | Terpene | 38.0 | 87.5 | 133.0 | 7.8e-31 |
| AAY28422.1 | geranylgeranyl\_diphosphate\_synthase | BGC0000630 | Terpene | 37.0 | 71.7 | 127.0 | 3.3e-29 |
| DAC74134.1 | GGPP\_synthase | BGC0002019 | Terpene | 34.0 | 75.8 | 118.0 | 1.5e-26 |
| BAA14124.1 |  | BGC0000638 | Terpene | 38.0 | 73.1 | 112.0 | 1.4e-24 |
| KZM73521.1 | hypothetical\_protein | BGC0000632 | Terpene + Saccharide | 33.0 | 77.8 | 111.0 | 3.2e-24 |
| WP\_091316341.1 | polyprenyl\_synthetase\_family\_protein | BGC0002007 | Terpene | 34.0 | 94.3 | 108.0 | 1.6e-23 |
| CCH63737.1 | XiaK\_protein | BGC0000665 | Terpene + Alkaloid | 36.0 | 72.7 | 108.0 | 2e-23 |
| AFK78080.1 | XiaN | BGC0000666 | Terpene | 35.0 | 72.7 | 105.0 | 1.7e-22 |
| ADO85577.1 | PntB | BGC0000653 | Terpene | 36.0 | 71.0 | 103.0 | 5e-22 |
| ABT18037.1 | heptaprenyl\_diphosphate\_synthase | BGC0000905 | Other | 31.0 | 89.6 | 92.0 | 1.5e-18 |
| BBC27537.1 | polyprenyl\_synthetase | BGC0001914 | Other | 30.0 | 84.5 | 90.0 | 4.4e-18 |
| ADD83016.1 | GGPP\_synthease | BGC0001156 | Terpene | 30.0 | 75.8 | 86.0 | 6.4e-17 |
| BAG23659.1 | putative\_geranylgeranyl\_pyrophosphate\_synthase | BGC0000664 | Terpene | 31.0 | 85.5 | 86.0 | 8.3e-17 |
| ABB69754.1 | PlaT4 | BGC0000654 | Terpene + Saccharide:Hybrid/tailoring | 32.0 | 76.4 | 85.0 | 1.9e-16 |
| ACO31283.1 | PtmT4 | BGC0001898 | Terpene | 30.0 | 75.8 | 84.0 | 4.1e-16 |
| AAG28699.1 | CrtE | BGC0000649 | Terpene | 30.0 | 85.5 | 83.0 | 5.4e-16 |
| AGZ20472.2 | geranylgeranyl\_diphosphate\_synthase | BGC0001776 | Terpene | 25.0 | 79.8 | 61.0 | 2.2e-09 |
| ctg1\_orf003 |  | BGC0000688 | Terpene | 25.0 | 85.5 | 61.0 | 3.8e-09 |
| ANV81299.1 | GGPP\_synthase | BGC0001604 | Terpene | 29.0 | 64.6 | 58.0 | 1.9e-08 |
| BAD29970.1 | geranylgeranyldiphosphate\_synthase | BGC0000676 | Terpene | 27.0 | 80.1 | 58.0 | 2.4e-08 |
| BBE36502.1 | putative\_bifunctional\_terpene\_synthase | BGC0001915 | Terpene | 25.0 | 70.7 | 51.0 | 2.3e-06 |
