# Supplementary material for: Genome of Methylomonas sp. AM2-LC, representing a methanotrophic bacterial species isolated from water column of a boreal, oxygen-stratified lake
Source: Front Genet. 2024 Aug 30;15:1440435. doi: 10.3389/fgene.2024.1440435 (PMC11392852; doi:10.3389/fgene.2024.1440435)
Supplement: Supplementary file 3 [file DataSheet2.ZIP › Supplementary_File_2/08.gene_cluster/AMSG_genome.zip!/knownclusterblast/region5/GE004015_mibig_hits.html]

| MIBiG Protein | Description | MIBiG Cluster | MiBiG Product | % ID | % Coverage | BLAST Score | E-value |
| --- | --- | --- | --- | --- | --- | --- | --- |
| CAB39702.1 | probable\_aminotransferase | BGC0000663 | Terpene | 47.0 | 99.6 | 446.0 | 4.9e-125 |
| CAH55650.1 | putative\_aminotransferase | BGC0000259 | Polyketide | 33.0 | 95.0 | 287.0 | 3.8e-77 |
| ABB69077.1 | putative\_3-acetyloctanal\_aminotransferase | BGC0000260 | Polyketide | 35.0 | 96.1 | 284.0 | 3.2e-76 |
| CAH55633.1 | putative\_aminotransferase | BGC0000258 | Polyketide | 33.0 | 98.0 | 277.0 | 3e-74 |
| WP\_044618982.1 | aminotransferase\_class\_III-fold\_pyridoxal\_phosphate-dependent\_enzyme | BGC0001791 | NRP | 33.0 | 90.0 | 228.0 | 1.6e-59 |
| AFH74310.1 | 4-aminobutyrate\_aminotransferase | BGC0001151 | Other:Non-NRP beta-lactam | 35.0 | 85.2 | 205.0 | 1.9e-52 |
| ADD82957.1 | BatP | BGC0001099 | NRP + Polyketide:Modular type I + Polyketide:Trans-AT type I | 31.0 | 91.8 | 196.0 | 1.2e-49 |
| AAY37658.1 | diaminobutyrate\_aminotransferase\_apoenzyme | BGC0000437 | NRP | 30.0 | 92.2 | 183.0 | 6e-46 |
| AHY86404.1 | putative\_diaminobutyrate-2-oxoglutarate\_transaminase\_protein | BGC0000329 | NRP | 29.0 | 94.4 | 182.0 | 1.3e-45 |
| ARF06207.1 | ornithine--oxo-acid\_transaminase | BGC0001593 | NRP | 33.0 | 77.2 | 181.0 | 3e-45 |
| ctg1\_orf1260 |  | BGC0001752 | NRP | 33.0 | 84.6 | 178.0 | 3.3e-44 |
| AEZ53937.1 | putative\_ornithine\_aminotransferase | BGC0000144 | Polyketide:Modular type I | 37.0 | 66.4 | 176.0 | 7.3e-44 |
| AFH75325.1 | diaminobutyrate-2-oxoglutarate\_aminotransferase | BGC0000425 | NRP:Cyclic depsipeptide | 29.0 | 94.1 | 171.0 | 4e-42 |
| AAD45546.1 | SpcC | BGC0000716 | Saccharide | 31.0 | 86.8 | 169.0 | 1.5e-41 |
| CAJ96477.1 | aminotransferase | BGC0000330 | NRP:NRP siderophore | 28.0 | 98.7 | 164.0 | 4.9e-40 |
| WP\_082045523.1 | aminotransferase\_class\_III-fold\_pyridoxal\_phosphate-dependent\_enzyme | BGC0002001 | NRP + Polyketide | 28.0 | 88.7 | 161.0 | 2.4e-39 |
| KFL51879.1 | 2,4-diaminobutyrate\_4-aminotransferase | BGC0001711 | NRP + Polyketide | 27.0 | 96.7 | 160.0 | 5.4e-39 |
| ABW87801.1 | SpcS1 | BGC0000715 | Saccharide | 32.0 | 73.8 | 160.0 | 9.2e-39 |
| ABA41503.1 | aminotransferase | BGC0000722 | Polyketide + Other:Cyclitol | 27.0 | 89.4 | 159.0 | 1.2e-38 |
| CAA68242.1 | acetylornithine\_aminotransferase | BGC0000895 | Other | 35.0 | 69.6 | 158.0 | 3.5e-38 |
| WP\_013310334.1 | aspartate\_aminotransferase\_family\_protein | BGC0001728 | NRP + Polyketide | 29.0 | 91.3 | 158.0 | 3.5e-38 |
| ABC67270.1 | putative\_aminotransferase | BGC0000723 | Saccharide | 27.0 | 89.4 | 156.0 | 1e-37 |
| CDN62024.1 | Omega-amino\_acid--pyruvate\_aminotransferase | BGC0001599 | NRP | 30.0 | 76.6 | 156.0 | 1.3e-37 |
| AAC25608.1 | unknown | BGC0000919 | Other | 31.0 | 73.5 | 155.0 | 2.3e-37 |
| ACL77548.1 | aminotransferase\_class-III | BGC0001891 | Other | 31.0 | 75.7 | 154.0 | 3.9e-37 |
| AAK12084.1 | EctB | BGC0000852 | Other | 29.0 | 88.9 | 152.0 | 1.5e-36 |
| ADN26241.1 | PLP-dependent\_aminotransferase | BGC0000951 | NRP | 28.0 | 88.3 | 152.0 | 1.5e-36 |
| ACB30331.1 | L-2,4-diaminobutyric\_acid\_aminotransferase | BGC0000859 | Other | 29.0 | 91.1 | 149.0 | 9.5e-36 |
| AAY96771.1 | L-2,4-diaminobutyrat\_transaminase | BGC0000854 | Other | 29.0 | 90.7 | 146.0 | 8.1e-35 |
| ADZ45332.1 | aminotransferase | BGC0001020 | NRP + Polyketide | 29.0 | 75.3 | 145.0 | 2.3e-34 |
| ABT18049.1 | glutamate-1-semialdehyde\_2,1-aminotransferase | BGC0000905 | Other | 32.0 | 67.0 | 136.0 | 8.4e-32 |
| ABU41515.1 | HemL | BGC0000906 | Other | 32.0 | 67.7 | 134.0 | 5.4e-31 |
| EYT83456.1 | ornithine\_aminotransferase | BGC0001213 | Polyketide | 27.0 | 81.8 | 132.0 | 1.6e-30 |
| ARE72410.1 | putative\_aminotransferase | BGC0001510 | Other | 29.0 | 67.2 | 126.0 | 1.5e-28 |
| WP\_013184465.1 | diaminobutyrate--2-oxoglutarate\_transaminase | BGC0001716 | NRP | 25.0 | 92.6 | 124.0 | 3.3e-28 |
| ABV97170.1 | aminotransferase\_class-III | BGC0000137 | Polyketide | 27.0 | 92.6 | 121.0 | 4.7e-27 |
| BAP34749.1 | glutamate-1-semialdehyde\_aminotransferase | BGC0000078 | Polyketide | 28.0 | 73.8 | 117.0 | 6.8e-26 |
| AHD05679.1 | putative\_non-ribosomal\_peptide\_ligase/\_polyketide\_synthase\_hybrid | BGC0000402 | NRP | 30.0 | 65.5 | 115.0 | 2.6e-25 |
| PPE\_RS11475 | aminotransferase\_class\_III-fold\_pyridoxal\_phosphate-dependent\_enzyme | BGC0001993 | NRP | 28.0 | 76.4 | 114.0 | 5.8e-25 |
| AID65222.1 | putative\_aspartate\_racemase | BGC0000335 | NRP | 29.0 | 76.8 | 113.0 | 9.9e-25 |
| WP\_013310342.1 | non-ribosomal\_peptide\_synthetase | BGC0001993 | NRP | 28.0 | 65.9 | 113.0 | 9.9e-25 |
| AHB82056.1 | non\_ribosomal\_peptide\_synthetase | BGC0001019 | NRP + Polyketide:Modular type I | 28.0 | 75.3 | 112.0 | 1.7e-24 |
| AAF08795.1 | MycA | BGC0001103 | NRP + Polyketide | 28.0 | 70.9 | 112.0 | 2.2e-24 |
| ABS74181.1 | bacillomycin\_D\_synthetase\_A\_ | BGC0001090 | Polyketide + NRP:Lipopeptide | 28.0 | 70.9 | 110.0 | 8.4e-24 |
| BAB69698.1 | iturin\_A\_synthetase\_A | BGC0001098 | NRP + Polyketide | 28.0 | 70.9 | 110.0 | 8.4e-24 |
| AHB82069.1 | non\_ribosomal\_peptide\_synthetase | BGC0001231 | NRP + Polyketide:Modular type I | 31.0 | 69.4 | 110.0 | 8.4e-24 |
| AVI26390.1 | polyketide\_synthase\_/\_nonribosomal\_peptide\_synthase\_hybrid | BGC0001800 | NRP + Polyketide | 28.0 | 69.4 | 104.0 | 4.6e-22 |
| AEU11006.1 | NpnB | BGC0001029 | NRP + Polyketide | 29.0 | 65.3 | 104.0 | 6e-22 |
| CAD17792.1 | probable\_non\_ribosomal\_peptide\_synthetase\_protein | BGC0001754 | NRP + Polyketide | 28.0 | 64.2 | 100.0 | 6.6e-21 |
| CCM44332.1 | PfaD\_family\_protein | BGC0001056 | NRP + Polyketide:Modular type I + Polyketide:PUFA synthase or related | 28.0 | 63.6 | 99.0 | 1.1e-20 |
| PHM26608.1 | glutamate-1-semialdehyde-2,1-aminomutase | BGC0001130 | NRP + Polyketide | 27.0 | 63.6 | 96.0 | 1.6e-19 |
| CBJ82075.1 | putative\_Glutamate-1-semialdehyde\_2,1-aminomutase | BGC0001872 | Polyketide | 26.0 | 63.6 | 96.0 | 1.6e-19 |
| WP\_051094164.1 | aminotransferase\_class\_III-fold\_pyridoxal\_phosphate-dependent\_enzyme | BGC0001332 | NRP + Polyketide | 25.0 | 64.6 | 95.0 | 2.1e-19 |
| BAF92604.1 | aminotransferase | BGC0000118 | Polyketide | 26.0 | 64.0 | 95.0 | 2.8e-19 |
| ACJ24878.1 | glutamate-1-semialdehyde\_aminotransferase-like\_protein | BGC0000119 | Polyketide:Iterative type I + Saccharide:Hybrid/tailoring | 26.0 | 64.0 | 95.0 | 2.8e-19 |
| AFD30959.1 | CrmG | BGC0000966 | NRP + Polyketide | 24.0 | 66.6 | 86.0 | 1.7e-16 |
| ABC35928.1 | glutamate-1-semialdehyde\_aminotransferase | BGC0000386 | NRP:NRP siderophore | 25.0 | 62.5 | 70.0 | 7.4e-12 |
