# Supplementary material for: Genome of Methylomonas sp. AM2-LC, representing a methanotrophic bacterial species isolated from water column of a boreal, oxygen-stratified lake
Source: Front Genet. 2024 Aug 30;15:1440435. doi: 10.3389/fgene.2024.1440435 (PMC11392852; doi:10.3389/fgene.2024.1440435)
Supplement: Supplementary file 3 [file DataSheet2.ZIP › Supplementary_File_2/08.gene_cluster/AMSG_genome.zip!/knownclusterblast/region5/GE004011_mibig_hits.html]

| MIBiG Protein | Description | MIBiG Cluster | MiBiG Product | % ID | % Coverage | BLAST Score | E-value |
| --- | --- | --- | --- | --- | --- | --- | --- |
| ABC35620.1 | radical\_SAM\_domain\_protein | BGC0000386 | NRP:NRP siderophore | 62.0 | 84.0 | 443.0 | 2.7e-124 |
| CAB39699.1 | conserved\_hypothetical\_protein | BGC0000663 | Terpene | 49.0 | 86.3 | 362.0 | 7.9e-100 |
