# Supplementary material for: Genome of Methylomonas sp. AM2-LC, representing a methanotrophic bacterial species isolated from water column of a boreal, oxygen-stratified lake
Source: Front Genet. 2024 Aug 30;15:1440435. doi: 10.3389/fgene.2024.1440435 (PMC11392852; doi:10.3389/fgene.2024.1440435)
Supplement: Supplementary file 3 [file DataSheet2.ZIP › Supplementary_File_2/08.gene_cluster/AMSG_genome.zip!/knownclusterblast/region5/GE004020_mibig_hits.html]

| MIBiG Protein | Description | MIBiG Cluster | MiBiG Product | % ID | % Coverage | BLAST Score | E-value |
| --- | --- | --- | --- | --- | --- | --- | --- |
| AAL26690.1 | unknown | BGC0000758 | Saccharide:Exopolysaccharide | 32.0 | 84.7 | 183.0 | 9.1e-46 |
| ADG87283.1 | amidohydrolase | BGC0001860 | RiPP:Thiopeptide | 31.0 | 93.7 | 163.0 | 5.7e-40 |
| AEM00622.1 | aminohydrolase/peptidase | BGC0000605 | RiPP:Thiopeptide | 34.0 | 65.0 | 139.0 | 8.8e-33 |
| ACS83788.1 | peptidase | BGC0000613 | RiPP:Thiopeptide | 31.0 | 85.4 | 132.0 | 1.4e-30 |
