# Supplementary material for: Genome of Methylomonas sp. AM2-LC, representing a methanotrophic bacterial species isolated from water column of a boreal, oxygen-stratified lake
Source: Front Genet. 2024 Aug 30;15:1440435. doi: 10.3389/fgene.2024.1440435 (PMC11392852; doi:10.3389/fgene.2024.1440435)
Supplement: Supplementary file 3 [file DataSheet2.ZIP › Supplementary_File_2/08.gene_cluster/AMSG_genome.zip!/knownclusterblast/region5/GE004013_mibig_hits.html]

| MIBiG Protein | Description | MIBiG Cluster | MiBiG Product | % ID | % Coverage | BLAST Score | E-value |
| --- | --- | --- | --- | --- | --- | --- | --- |
| AXA20105.1 | squalene--hopene\_cyclase | BGC0001946 | NRP + Polyketide | 48.0 | 98.9 | 605.0 | 9e-173 |
| CAB39697.1 | putative\_squalene-hopene\_cyclase | BGC0000663 | Terpene | 36.0 | 94.9 | 402.0 | 1.5e-111 |
| chr3.CM0292.40.r2.m |  | BGC0001317 | Terpene | 25.0 | 102.8 | 172.0 | 2.5e-42 |
| ACF70484.1 | oxidosqualene\_clavarinone\_cyclase | BGC0001248 | Terpene | 25.0 | 100.3 | 139.0 | 1.8e-32 |
| ABB69743.1 | PlaT2 | BGC0000654 | Terpene + Saccharide:Hybrid/tailoring | 24.0 | 41.4 | 76.0 | 1.5e-13 |
