# Supplementary material for: Genome of Methylomonas sp. AM2-LC, representing a methanotrophic bacterial species isolated from water column of a boreal, oxygen-stratified lake
Source: Front Genet. 2024 Aug 30;15:1440435. doi: 10.3389/fgene.2024.1440435 (PMC11392852; doi:10.3389/fgene.2024.1440435)
Supplement: Supplementary file 3 [file DataSheet2.ZIP › Supplementary_File_2/08.gene_cluster/AMSG_genome.zip!/knownclusterblast/region5/GE004005_mibig_hits.html]

| MIBiG Protein | Description | MIBiG Cluster | MiBiG Product | % ID | % Coverage | BLAST Score | E-value |
| --- | --- | --- | --- | --- | --- | --- | --- |
| AVL27075.1 | tryptophanyl-tRNA\_synthetase | BGC0001546 | Alkaloid | 35.0 | 94.2 | 155.0 | 2.2e-37 |
| AJT38681.1 | Ind0 | BGC0001206 | Other | 33.0 | 93.0 | 154.0 | 3.8e-37 |
| ACB47039.1 | putative\_tryptophanyl-tRNA\_synthetase | BGC0001060 | Polyketide | 32.0 | 93.3 | 148.0 | 2.7e-35 |
| CAF31422.1 | putative\_Trp-tRNA\_ligase | BGC0000696 | Saccharide | 31.0 | 98.8 | 144.0 | 3e-34 |
| ARV75699.1 | putative\_tryptophanyl-tRNA\_synthatase | BGC0001603 | Saccharide | 31.0 | 98.8 | 144.0 | 3e-34 |
| CAF34046.1 | putative\_tryptophanyl-tRNA\_synthatase | BGC0000689 | Saccharide | 31.0 | 98.8 | 142.0 | 1.5e-33 |
| ATE50867.1 | tryptophanyl-tRNA\_synthetase | BGC0001485 | Other | 34.0 | 84.2 | 134.0 | 3.1e-31 |
