# Supplementary material for: Genome of Methylomonas sp. AM2-LC, representing a methanotrophic bacterial species isolated from water column of a boreal, oxygen-stratified lake
Source: Front Genet. 2024 Aug 30;15:1440435. doi: 10.3389/fgene.2024.1440435 (PMC11392852; doi:10.3389/fgene.2024.1440435)
Supplement: Supplementary file 3 [file DataSheet2.ZIP › Supplementary_File_2/08.gene_cluster/AMSG_genome.zip!/knownclusterblast/region5/GE004018_mibig_hits.html]

| MIBiG Protein | Description | MIBiG Cluster | MiBiG Product | % ID | % Coverage | BLAST Score | E-value |
| --- | --- | --- | --- | --- | --- | --- | --- |
| AGO55051.1 | 1-deoxy-D-xylulose-5-phosphate\_synthase\_Dxs | BGC0001361 | Terpene | 56.0 | 97.4 | 671.0 | 9.7e-193 |
| ATN39902.1 | MstH | BGC0001664 | Terpene | 49.0 | 100.2 | 565.0 | 9.8e-161 |
| APZ79619.1 | FisE4 | BGC0001595 | Alkaloid | 48.0 | 100.0 | 563.0 | 4.9e-160 |
| APZ79598.1 | FilE4 | BGC0001501 | Alkaloid | 48.0 | 100.3 | 561.0 | 1.4e-159 |
| APZ79565.1 | FimE4 | BGC0001594 | Alkaloid | 48.0 | 100.3 | 561.0 | 1.4e-159 |
| AHB62775.1 | 1-deoxy-D-xylulose-5-phosphate\_synthase\_(DXS) | BGC0001126 | Terpene + Alkaloid | 48.0 | 100.3 | 561.0 | 1.8e-159 |
| APB62261.1 | 1-deoxy-D-xylulose-5-phosphate\_synthase | BGC0001612 | Alkaloid | 48.0 | 100.3 | 561.0 | 1.8e-159 |
| AHI58837.1 | 1-deoxy-D-xylulose-5-phosphate\_synthase\_(DXS) | BGC0000668 | Terpene + Alkaloid | 48.0 | 100.3 | 560.0 | 4.1e-159 |
| CAB39701.1 | probable\_transketolase | BGC0000663 | Terpene | 40.0 | 100.3 | 436.0 | 6.9e-122 |
| ABB69756.1 | PlaT6 | BGC0000654 | Terpene + Saccharide:Hybrid/tailoring | 39.0 | 99.5 | 411.0 | 1.8e-114 |
| ADD83001.1 | DXP\_synthase | BGC0001156 | Terpene | 39.0 | 100.3 | 408.0 | 2.6e-113 |
| AFK78071.1 | XiaE | BGC0000666 | Terpene | 38.0 | 99.7 | 404.0 | 2.9e-112 |
| CCH63728.1 | XiaB\_protein | BGC0000665 | Terpene + Alkaloid | 38.0 | 99.7 | 403.0 | 4.9e-112 |
| ACO31286.1 | PtmM3 | BGC0001898 | Terpene | 38.0 | 99.7 | 400.0 | 4.2e-111 |
| BAF98621.1 | putative\_1-deoxy-D-xylulose-5-phosphate\_synthase | BGC0000651 | Terpene | 34.0 | 96.0 | 304.0 | 3.1e-82 |
