# Supplementary material for: Genome of Methylomonas sp. AM2-LC, representing a methanotrophic bacterial species isolated from water column of a boreal, oxygen-stratified lake
Source: Front Genet. 2024 Aug 30;15:1440435. doi: 10.3389/fgene.2024.1440435 (PMC11392852; doi:10.3389/fgene.2024.1440435)
Supplement: Supplementary file 3 [file DataSheet2.ZIP › Supplementary_File_2/08.gene_cluster/AMSG_genome.zip!/knownclusterblast/region5/GE004012_mibig_hits.html]

| MIBiG Protein | Description | MIBiG Cluster | MiBiG Product | % ID | % Coverage | BLAST Score | E-value |
| --- | --- | --- | --- | --- | --- | --- | --- |
| gene2 |  | BGC0001839 | Terpene | 30.0 | 80.7 | 99.0 | 1.5e-20 |
| ACM07426.1 | CrtM | BGC0000645 | Terpene | 26.0 | 67.8 | 63.0 | 7.1e-10 |
| CAA79957.1 | phytoene\_synthetase | BGC0000648 | Terpene | 31.0 | 29.8 | 52.0 | 2.1e-06 |
