# Supplementary material for: Genome of Methylomonas sp. AM2-LC, representing a methanotrophic bacterial species isolated from water column of a boreal, oxygen-stratified lake
Source: Front Genet. 2024 Aug 30;15:1440435. doi: 10.3389/fgene.2024.1440435 (PMC11392852; doi:10.3389/fgene.2024.1440435)
Supplement: Supplementary file 3 [file DataSheet2.ZIP › Supplementary_File_2/08.gene_cluster/AMSG_genome.zip!/knownclusterblast/region4/GE003112_mibig_hits.html]

| MIBiG Protein | Description | MIBiG Cluster | MiBiG Product | % ID | % Coverage | BLAST Score | E-value |
| --- | --- | --- | --- | --- | --- | --- | --- |
| ABX02681.1 | heat\_shock\_protein\_DnaJ\_domain\_protein | BGC0001521 | NRP | 36.0 | 104.8 | 180.0 | 4.5e-45 |
