# Supplementary material for: Genome of Methylomonas sp. AM2-LC, representing a methanotrophic bacterial species isolated from water column of a boreal, oxygen-stratified lake
Source: Front Genet. 2024 Aug 30;15:1440435. doi: 10.3389/fgene.2024.1440435 (PMC11392852; doi:10.3389/fgene.2024.1440435)
Supplement: Supplementary file 3 [file DataSheet2.ZIP › Supplementary_File_2/08.gene_cluster/AMSG_genome.zip!/knownclusterblast/region4/GE003110_mibig_hits.html]

| MIBiG Protein | Description | MIBiG Cluster | MiBiG Product | % ID | % Coverage | BLAST Score | E-value |
| --- | --- | --- | --- | --- | --- | --- | --- |
| WP\_006494985.1 | Crp/Fnr\_family\_transcriptional\_regulator | BGC0001589 | Saccharide | 37.0 | 95.4 | 155.0 | 1.5e-37 |
| WP\_004189279.1 | Crp/Fnr\_family\_transcriptional\_regulator | BGC0001588 | Saccharide | 38.0 | 91.6 | 151.0 | 1.7e-36 |
| WP\_004189328.1 | Crp/Fnr\_family\_transcriptional\_regulator | BGC0001588 | Saccharide | 31.0 | 94.1 | 127.0 | 3.4e-29 |
| WP\_043033125.1 | Crp/Fnr\_family\_transcriptional\_regulator | BGC0001589 | Saccharide | 30.0 | 95.8 | 120.0 | 3.2e-27 |
