# Supplementary material for: Genome of Methylomonas sp. AM2-LC, representing a methanotrophic bacterial species isolated from water column of a boreal, oxygen-stratified lake
Source: Front Genet. 2024 Aug 30;15:1440435. doi: 10.3389/fgene.2024.1440435 (PMC11392852; doi:10.3389/fgene.2024.1440435)
Supplement: Supplementary file 3 [file DataSheet2.ZIP › Supplementary_File_2/08.gene_cluster/AMSG_genome.zip!/knownclusterblast/region4/GE003108_mibig_hits.html]

| MIBiG Protein | Description | MIBiG Cluster | MiBiG Product | % ID | % Coverage | BLAST Score | E-value |
| --- | --- | --- | --- | --- | --- | --- | --- |
| AXA20105.1 | squalene--hopene\_cyclase | BGC0001946 | NRP + Polyketide | 48.0 | 92.4 | 609.0 | 5.2e-174 |
| CAB39697.1 | putative\_squalene-hopene\_cyclase | BGC0000663 | Terpene | 33.0 | 87.0 | 343.0 | 6.9e-94 |
| chr3.CM0292.40.r2.m |  | BGC0001317 | Terpene | 24.0 | 95.0 | 163.0 | 9.8e-40 |
| NP\_198464.4 | putative\_pentacyclic\_triterpene\_synthase\_3 | BGC0001314 | Terpene | 22.0 | 92.9 | 148.0 | 3.3e-35 |
| NP\_001318680.1 | putative\_pentacyclic\_triterpene\_synthase\_3 | BGC0001314 | Terpene | 22.0 | 92.9 | 148.0 | 3.3e-35 |
| NP\_001078733.1 | thalianol\_synthase\_1 | BGC0000670 | Terpene | 23.0 | 92.8 | 144.0 | 6.1e-34 |
| NP\_001329547.1 | baruol\_synthase\_1 | BGC0001313 | Terpene | 23.0 | 92.2 | 143.0 | 1.4e-33 |
| NP\_199074.1 | marneral\_synthase | BGC0000669 | Terpene | 22.0 | 92.2 | 142.0 | 2.3e-33 |
| NP\_193272.1 | baruol\_synthase\_1 | BGC0001313 | Terpene | 23.0 | 92.6 | 142.0 | 3e-33 |
| NP\_199612.3 | thalianol\_synthase\_1 | BGC0000670 | Terpene | 23.0 | 92.4 | 139.0 | 2e-32 |
| NP\_567462.1 | pentacyclic\_triterpene\_synthase\_1 | BGC0001313 | Terpene | 23.0 | 92.9 | 136.0 | 1.3e-31 |
