# Supplementary material for: Genome of Methylomonas sp. AM2-LC, representing a methanotrophic bacterial species isolated from water column of a boreal, oxygen-stratified lake
Source: Front Genet. 2024 Aug 30;15:1440435. doi: 10.3389/fgene.2024.1440435 (PMC11392852; doi:10.3389/fgene.2024.1440435)
Supplement: Supplementary file 3 [file DataSheet2.ZIP › Supplementary_File_2/08.gene_cluster/AMSG_genome.zip!/knownclusterblast/region4/GE003118_mibig_hits.html]

| MIBiG Protein | Description | MIBiG Cluster | MiBiG Product | % ID | % Coverage | BLAST Score | E-value |
| --- | --- | --- | --- | --- | --- | --- | --- |
| AWM72908.1 | ABC-transporter\_(ATP-binding) | BGC0001719 | Polyketide | 30.0 | 91.9 | 93.0 | 5.7e-19 |
| AEX55154.1 | putative\_lantibiotic\_transport\_ATP-binding\_protein\_SlvF | BGC0000549 | RiPP:Lanthipeptide | 27.0 | 92.7 | 92.0 | 7.4e-19 |
| AHL46714.1 | ABc\_transporter\_protein | BGC0001179 | Polyketide:Type II | 29.0 | 94.7 | 92.0 | 1.3e-18 |
| CAE02640.1 | YckI\_protein | BGC0000433 | NRP:Lipopeptide | 30.0 | 87.4 | 91.0 | 1.7e-18 |
| ABC37732.1 | putrescine\_ABC\_transporter,\_ATP-binding\_protein | BGC0001120 | NRP + Polyketide:Modular type I | 31.0 | 89.5 | 91.0 | 2.8e-18 |
| CAK15811.1 | putative\_polyamine\_ABC\_transporter,\_ATP\_binding\_protein | BGC0000344 | NRP | 27.0 | 98.8 | 90.0 | 4.8e-18 |
| AOT85876.1 | immunity\_protein | BGC0001789 | RiPP | 27.0 | 97.2 | 90.0 | 4.8e-18 |
| ABO65658.1 | Putative\_ABC\_transporter\_ATP-binding\_protein | BGC0000515 | RiPP:Lanthipeptide | 26.0 | 98.0 | 89.0 | 6.3e-18 |
| AHN91916.1 | ABC\_transporter | BGC0000340 | NRP | 28.0 | 90.3 | 87.0 | 3.1e-17 |
| ASY03206.1 | NsoF | BGC0001701 | RiPP | 28.0 | 92.3 | 87.0 | 4.1e-17 |
| ABA00875.1 | NsuF | BGC0000537 | RiPP:Lanthipeptide | 29.0 | 82.2 | 86.0 | 9.1e-17 |
| AAL15571.1 | putative\_SpaF | BGC0000511 | RiPP:Lanthipeptide | 27.0 | 85.8 | 84.0 | 3.4e-16 |
| AAC72253.1 | LctF | BGC0000521 | RiPP:Lanthipeptide | 29.0 | 89.9 | 82.0 | 7.7e-16 |
| AAB91597.1 | SpaF | BGC0000559 | RiPP:Lanthipeptide | 26.0 | 85.8 | 80.0 | 3.8e-15 |
| CAQ64719.1 | sugar\_uptake\_ABC\_transporter,\_ATP-binding\_protein | BGC0000087 | Polyketide | 28.0 | 90.7 | 79.0 | 6.5e-15 |
| AAU39356.1 | putative\_spermidine/putrescine\_ABC\_transporter\_ATP-binding\_protein | BGC0000381 | NRP | 26.0 | 98.0 | 79.0 | 8.5e-15 |
| AEK64496.1 | EtnF | BGC0000506 | RiPP:Lanthipeptide | 26.0 | 85.8 | 79.0 | 1.1e-14 |
| BAG71487.1 | immunity\_protein | BGC0000536 | RiPP:Lanthipeptide | 29.0 | 89.1 | 79.0 | 1.1e-14 |
| AUD08667.1 | ABC\_transporter\_ATP-binding\_protein | BGC0001553 | NRP + Polyketide | 27.0 | 93.9 | 79.0 | 1.1e-14 |
| AQP25584.1 | glutamate\_ABC\_transporter | BGC0001590 | Polyketide | 26.0 | 98.0 | 79.0 | 1.1e-14 |
| ACR33047.1 | ABC\_transport\_protein\_ATP-binding | BGC0000495 | RiPP:Lanthipeptide | 26.0 | 93.5 | 78.0 | 1.4e-14 |
| CAL82046.1 | putative\_cytolysin\_ABC\_transporter,\_ATP-binding\_protein | BGC0000564 | RiPP:LAP | 29.0 | 92.7 | 77.0 | 2.5e-14 |
| AAK33690.1 | ABC\_transporter\_ | BGC0000566 | RiPP:LAP | 29.0 | 92.7 | 77.0 | 2.5e-14 |
| ADJ56360.1 | NisF | BGC0000535 | RiPP:Lanthipeptide | 27.0 | 88.7 | 76.0 | 7.2e-14 |
| ALD83698.1 | ABC\_transporter\_ATP\_binding\_protein | BGC0001299 | Polyketide | 28.0 | 86.6 | 75.0 | 1.6e-13 |
| AVH76806.1 | BcrA | BGC0001669 | RiPP | 33.0 | 69.2 | 75.0 | 1.6e-13 |
| ABM21744.1 | PdmR1 | BGC0000256 | Polyketide | 26.0 | 92.3 | 74.0 | 3.6e-13 |
| EHK80174.1 | ABC\_transporter\_ATP-binding\_protein | BGC0001447 | Polyketide | 28.0 | 89.5 | 73.0 | 4.7e-13 |
| CUX79067.1 | Bicarbonate\_transport\_ATP-binding\_protein\_CmpC | BGC0001715 | NRP | 31.0 | 80.2 | 73.0 | 4.7e-13 |
| BAK64654.1 | putative\_transporter | BGC0000135 | Polyketide | 28.0 | 89.1 | 73.0 | 6.1e-13 |
| WP\_042386113.1 | ABC\_transporter\_ATP-binding\_protein | BGC0001170 | RiPP:LAP | 28.0 | 93.1 | 73.0 | 6.1e-13 |
| WP\_003211121.1 | ABC\_transporter\_ATP-binding\_protein | BGC0001173 | RiPP:LAP | 28.0 | 90.7 | 72.0 | 1e-12 |
| YP\_002539385.1 | SitB | BGC0001555 | RiPP | 28.0 | 91.9 | 72.0 | 1e-12 |
| ANH11417.1 | SceV | BGC0001908 | Polyketide | 26.0 | 93.1 | 72.0 | 1e-12 |
| AAY37657.1 | ABC\_transporter:Protein\_of\_unknown\_function\_DUF214 | BGC0000437 | NRP | 31.0 | 84.6 | 72.0 | 1.4e-12 |
| AHF21240.1 | CrnT | BGC0000500 | RiPP:Lanthipeptide | 33.0 | 64.4 | 72.0 | 1.4e-12 |
| AHZ34249.1 | MacB | BGC0001389 | NRP | 27.0 | 100.4 | 72.0 | 1.4e-12 |
| BAC98762.1 | NukF | BGC0000539 | RiPP:Lanthipeptide | 26.0 | 97.2 | 71.0 | 2.3e-12 |
| CAB11989.1 | sporulation\_killing\_factor\_biosynthesis\_and\_export;\_ABC\_transporter\_(binding\_protein) | BGC0000601 | RiPP:Head-to-tailcyclized peptide | 29.0 | 70.9 | 71.0 | 2.3e-12 |
| AHY86401.1 | ABC\_transporter\_protein | BGC0000329 | NRP | 27.0 | 106.5 | 71.0 | 3e-12 |
| AJW76714.1 | DsaL | BGC0001196 | NRP | 28.0 | 85.0 | 71.0 | 3e-12 |
| CAD91242.1 | hypothetical\_protein | BGC0000289 | NRP:Glycopeptide + Saccharide:Hybrid/tailoring | 26.0 | 92.7 | 70.0 | 3.9e-12 |
| ALA09377.1 | ABC\_transporter | BGC0001303 | Polyketide | 26.0 | 80.6 | 70.0 | 3.9e-12 |
| AAF81232.1 | ABC\_transporter\_ATP\_binding\_protein | BGC0000243 | Polyketide | 24.0 | 95.1 | 69.0 | 6.7e-12 |
| AFH75332.1 | macrolide\_export\_ATP-binding/permease\_protein\_MacB | BGC0000398 | NRP:Cyclic depsipeptide | 28.0 | 105.3 | 69.0 | 6.7e-12 |
| AFW17839.1 | PaeF | BGC0000541 | RiPP:Lanthipeptide | 25.0 | 89.9 | 69.0 | 8.8e-12 |
| AEA03264.1 | ABC\_transporter\_ATP-binding\_subunit | BGC0000563 | RiPP:Lanthipeptide | 25.0 | 88.7 | 69.0 | 8.8e-12 |
| ANZ15844.1 | putative\_ABC\_transporter\_ATP-binding\_protein | BGC0001569 | NRP | 27.0 | 86.2 | 69.0 | 1.1e-11 |
| AFH75324.1 | macrolide\_export\_ATP-binding/permease\_protein\_MacB | BGC0000425 | NRP:Cyclic depsipeptide | 29.0 | 80.2 | 68.0 | 1.5e-11 |
| AFU82624.1 | ABC\_transporter,\_ATPase\_component | BGC0000998 | NRP + Polyketide | 27.0 | 86.6 | 68.0 | 1.5e-11 |
| AJO72722.1 | Antibiotic\_ABC\_transporter\_ATPase | BGC0001381 | Polyketide | 27.0 | 87.0 | 68.0 | 1.5e-11 |
| ABA73958.1 | macrolide-specific\_ABC-type\_efflux\_carrier | BGC0001842 | NRP:Lipopeptide | 28.0 | 88.3 | 68.0 | 1.5e-11 |
| ABP55202.1 | ABC\_transporter\_related | BGC0000142 | Polyketide | 26.0 | 80.6 | 68.0 | 2e-11 |
| CAB93668.2 | RumF\_protein | BGC0000545 | RiPP:Lanthipeptide | 27.0 | 90.3 | 68.0 | 2e-11 |
| orf4 | ABC\_transporter\_ATP-binding\_protein | BGC0001429 | NRP:Cyclic depsipeptide + Polyketide:Iterative type I | 28.0 | 95.1 | 62.0 | 8.2e-10 |
| APZ78843.1 | ABC\_transporter\_ATP-binding\_protein | BGC0001431 | NRP:Cyclic depsipeptide + Polyketide:Iterative type I | 30.0 | 96.8 | 68.0 | 2e-11 |
| ACL77551.1 | ABC\_transporter\_related | BGC0001891 | Other | 24.0 | 91.1 | 67.0 | 3.3e-11 |
| ctg1\_orf15 |  | BGC0000053 | Polyketide | 27.0 | 90.3 | 67.0 | 4.4e-11 |
| CAY48791.1 | macrolide-specific\_ABC-type\_efflux\_carrier | BGC0001312 | NRP | 30.0 | 90.3 | 67.0 | 4.4e-11 |
| CCB53261.1 | ABC\_transporter\_family\_protein | BGC0001393 | NRP | 26.0 | 92.7 | 67.0 | 4.4e-11 |
| AOT85882.1 | immunity\_protein | BGC0001787 | RiPP | 23.0 | 90.3 | 67.0 | 4.4e-11 |
| CCJ67642.1 | macrolide\_export\_ATP-binding/permease\_protein\_MacB | BGC0000447 | NRP:Lipopeptide | 28.0 | 82.2 | 66.0 | 5.7e-11 |
| ADO17962.1 | CclG | BGC0000487 | RiPP:Head-to-tailcyclized peptide | 27.0 | 84.6 | 66.0 | 5.7e-11 |
| AOA33125.1 | ABC\_transporter\_ATP-binding\_protein | BGC0001346 | NRP:Cyclic depsipeptide | 28.0 | 87.4 | 66.0 | 5.7e-11 |
| ABP54626.1 | ABC\_transporter\_related | BGC0000241 | Polyketide:Type II + Saccharide:Hybrid/tailoring | 32.0 | 65.2 | 66.0 | 7.4e-11 |
| CAN02020.1 | putative\_ABC\_transporter,\_ATP-binding\_protein | BGC0000528 | RiPP:Lanthipeptide | 31.0 | 71.7 | 66.0 | 7.4e-11 |
| CCQ18698.1 | ABC\_transporter\_ATP-binding\_domain | BGC0000544 | RiPP:Lanthipeptide | 30.0 | 87.4 | 66.0 | 7.4e-11 |
| KUM69048.1 | ABC\_transporter\_ATP-binding\_protein | BGC0001561 | NRP | 26.0 | 85.0 | 66.0 | 7.4e-11 |
| AAZ76598.1 | BhtF | BGC0000497 | RiPP:Lanthipeptide | 29.0 | 89.5 | 66.0 | 9.7e-11 |
| AHF21231.1 | PabF | BGC0000542 | RiPP:Lanthipeptide | 23.0 | 90.7 | 66.0 | 9.7e-11 |
| ADH04633.1 | unknown | BGC0001051 | NRP + Polyketide:Modular type I | 28.0 | 84.2 | 66.0 | 9.7e-11 |
| CCA65705.1 | predicted\_ABC\_transporter\_ATP-binding\_protein | BGC0001343 | Polyketide | 29.0 | 80.2 | 66.0 | 9.7e-11 |
| AAY93343.1 | efflux\_ABC\_transporter,\_ATP-binding/permease\_protein | BGC0000413 | NRP | 27.0 | 88.7 | 65.0 | 1.3e-10 |
| BAD72772.1 |  | BGC0000552 | RiPP:Lanthipeptide | 28.0 | 91.9 | 65.0 | 1.3e-10 |
| AAP03101.1 | putative\_ATP-binding\_protein | BGC0000874 | Other | 28.0 | 101.2 | 65.0 | 1.3e-10 |
| ABW17379.1 | MacB | BGC0000411 | NRP | 28.0 | 87.9 | 65.0 | 1.7e-10 |
| ATU31790.1 | ABC\_transporter\_ATP-binding\_protein | BGC0001814 | NRP | 26.0 | 84.2 | 65.0 | 1.7e-10 |
| QCE20595.1 | ABC\_transporter | BGC0001961 | NRP | 25.0 | 80.2 | 65.0 | 1.7e-10 |
| AAY91423.1 | efflux\_ABC\_transporter,\_permease/ATP-binding\_protein | BGC0000399 | NRP:Cyclic depsipeptide | 26.0 | 99.6 | 64.0 | 2.2e-10 |
| ALA09343.1 | hypothetical\_protein | BGC0001303 | Polyketide | 25.0 | 90.3 | 64.0 | 2.2e-10 |
| ARD70852.1 | ABC\_transporter | BGC0001693 | Polyketide | 28.0 | 73.3 | 64.0 | 2.2e-10 |
| QDF82252.1 | macrolide\_export\_ATP-binding/permease\_protein | BGC0001980 | NRP | 28.0 | 80.6 | 64.0 | 2.2e-10 |
| G339\_RS33385 | ABC\_transporter\_ATP-binding\_protein | BGC0001364 | RiPP | 26.0 | 89.9 | 64.0 | 2.8e-10 |
| WP\_064118557.1 | MacB\_family\_efflux\_pump\_subunit | BGC0001509 | NRP | 28.0 | 87.9 | 64.0 | 2.8e-10 |
| CAK15812.1 | macrolide\_ABC\_efflux\_protein\_MacB | BGC0000344 | NRP | 29.0 | 80.2 | 64.0 | 3.7e-10 |
| BAC87901.1 | probable\_ferric\_acinetobactin\_transport\_system\_ATP-binding\_protein | BGC0000294 | NRP | 25.0 | 95.5 | 63.0 | 4.8e-10 |
| BAE95596.1 | putative\_sugar-transport\_ATP\_binding\_protein | BGC0000703 | Saccharide | 26.0 | 84.2 | 63.0 | 4.8e-10 |
| CAF60513.1 | putative\_sugar\_ABC-transproter\_ATP-binding\_protein | BGC0000704 | Saccharide | 26.0 | 84.2 | 63.0 | 4.8e-10 |
| BBC83962.1 | P-loop\_containing\_nucleoside\_triphosphate\_hydrolase\_protein | BGC0001636 | NRP | 23.0 | 90.7 | 63.0 | 4.8e-10 |
| WP\_017534155.1 | daunorubicin\_resistance\_protein\_DrrA\_family\_ABC\_transporter\_ATP-binding\_protein | BGC0001655 | RiPP | 25.0 | 92.7 | 63.0 | 4.8e-10 |
| AJW76720.1 | ATP-binding\_protein | BGC0001196 | NRP | 26.0 | 92.7 | 63.0 | 6.3e-10 |
| AFJ23828.1 | MacB-like\_ABC\_transporter\_protein | BGC0001838 | NRP | 27.0 | 87.9 | 63.0 | 6.3e-10 |
| CAI94676.1 | putative\_ABC\_transporter | BGC0000141 | Polyketide | 28.0 | 88.3 | 62.0 | 8.2e-10 |
| APZ78819.1 | ABC\_transporter\_ATP-binding\_protein | BGC0001429 | NRP:Cyclic depsipeptide + Polyketide:Iterative type I | 28.0 | 95.1 | 62.0 | 8.2e-10 |
| CAF31568.1 | putative\_sugar\_ABC\_transporter | BGC0000705 | Saccharide | 26.0 | 78.1 | 62.0 | 1.1e-09 |
| AHZ61838.1 | ABC\_transporter-like\_protein | BGC0000240 | Polyketide:Type II + Saccharide:Hybrid/tailoring | 30.0 | 65.2 | 62.0 | 1.4e-09 |
| AHZ34236.1 | MacB | BGC0000323 | NRP:Lipopeptide | 27.0 | 89.5 | 62.0 | 1.4e-09 |
| AEI58864.1 | ABC\_transporter | BGC0000455 | NRP | 27.0 | 91.5 | 62.0 | 1.4e-09 |
| AWR88445.1 | hypothetical\_protein | BGC0001522 | Polyketide | 28.0 | 73.7 | 61.0 | 1.8e-09 |
| CBA63688.1 | ATPase\_of\_griseobactin\_uptake\_ABC\_transporter | BGC0000368 | NRP | 26.0 | 93.5 | 61.0 | 3.1e-09 |
| ADK32558.1 | ABC\_transporter\_ATP-binding\_domain | BGC0000529 | RiPP:Lanthipeptide | 26.0 | 93.1 | 61.0 | 3.1e-09 |
| ACG70837.1 | ATP-binding\_phosphonate\_ABC\_transporter\_PhnC | BGC0000938 | Other:Aminocoumarin | 26.0 | 88.7 | 61.0 | 3.1e-09 |
| WP\_003948917.1 | ATP-binding\_cassette\_domain-containing\_protein | BGC0001792 | NRP | 26.0 | 89.1 | 61.0 | 3.1e-09 |
| ADK32548.1 | putative\_ABC\_transporter\_ATP-binding\_protein | BGC0000529 | RiPP:Lanthipeptide | 32.0 | 66.0 | 60.0 | 4.1e-09 |
| BAB08166.1 | SrtF | BGC0000556 | RiPP:Lanthipeptide | 24.0 | 76.1 | 60.0 | 4.1e-09 |
| ALV86877.1 | Tlo31 | BGC0001406 | NRP | 27.0 | 73.7 | 60.0 | 4.1e-09 |
| ADU86007.1 | putative\_ABC\_transporter-related\_protein | BGC0000165 | Polyketide:Modular type I | 28.0 | 85.8 | 60.0 | 5.3e-09 |
| ABD65952.1 | ABC\_transporter | BGC0000341 | NRP | 26.0 | 88.3 | 60.0 | 5.3e-09 |
| AIE77056.1 | ABC-transporter | BGC0000418 | NRP | 27.0 | 91.1 | 60.0 | 5.3e-09 |
| ALD83705.1 | ATP\_binding\_protein | BGC0001299 | Polyketide | 27.0 | 89.1 | 60.0 | 5.3e-09 |
| ADC96668.1 | ABC\_transporter | BGC0001076 | Other:Nucleoside | 28.0 | 92.7 | 59.0 | 7e-09 |
| BAG84245.1 | putative\_ABC-transporter | BGC0000257 | Polyketide | 27.0 | 82.6 | 59.0 | 9.1e-09 |
| AIG79244.1 | Conserved\_putative\_membrane\_protein | BGC0000419 | Saccharide + NRP:Glycopeptide | 27.0 | 90.7 | 59.0 | 9.1e-09 |
| EME52991.1 | ABC\_transporter | BGC0001460 | NRP:Glycopeptide | 27.0 | 91.5 | 59.0 | 9.1e-09 |
| OLZ50884.1 | ABC\_transporter | BGC0001461 | NRP:Glycopeptide | 28.0 | 92.3 | 59.0 | 9.1e-09 |
| YP\_002539412.1 | EtsB | BGC0001555 | RiPP | 26.0 | 76.9 | 59.0 | 9.1e-09 |
| AYA22337.1 | Tri | BGC0001955 | Other | 28.0 | 92.3 | 59.0 | 9.1e-09 |
| CAE53357.1 | ABC\_transporter | BGC0000440 | NRP:Glycopeptide | 28.0 | 91.5 | 59.0 | 1.2e-08 |
| CAG15016.1 | ABC\_transporter | BGC0000441 | NRP | 28.0 | 91.5 | 59.0 | 1.2e-08 |
| CAJ81021.1 | ABC\_transporter\_ATP-binding\_protein\_AcbW | BGC0000691 | Saccharide | 24.0 | 91.1 | 59.0 | 1.2e-08 |
| CAG38700.1 | putative\_ABC-transporter,\_ATP-binding\_protein | BGC0000708 | Saccharide | 27.0 | 90.3 | 59.0 | 1.2e-08 |
| AGS77319.1 | ABC\_transporter | BGC0001178 | NRP:Glycopeptide | 27.0 | 92.3 | 59.0 | 1.2e-08 |
| CTQ34884.1 | AtcG;\_putative\_ABC\_transporter | BGC0001301 | Polyketide | 25.0 | 86.6 | 59.0 | 1.2e-08 |
| WP\_013310325.1 | ABC\_transporter\_ATP-binding\_protein | BGC0001728 | NRP + Polyketide | 26.0 | 84.2 | 59.0 | 1.2e-08 |
| AGL93176.1 | ABC\_transporter\_ATP-binding\_protein | BGC0000560 | RiPP:Lanthipeptide | 28.0 | 96.4 | 58.0 | 1.5e-08 |
| OKA09422.1 | ABC\_transporter | BGC0001459 | NRP:Glycopeptide | 27.0 | 91.5 | 58.0 | 2e-08 |
| AUD11999.1 | OrbC | BGC0001721 | NRP | 25.0 | 102.0 | 58.0 | 2e-08 |
| CAD91214.1 | putative\_ABC\_transporter,\_Dbv19 | BGC0000289 | NRP:Glycopeptide + Saccharide:Hybrid/tailoring | 26.0 | 91.9 | 57.0 | 2.6e-08 |
| CAD91219.1 | putative\_ABC\_transporter,\_Dbv24 | BGC0000289 | NRP:Glycopeptide + Saccharide:Hybrid/tailoring | 27.0 | 91.5 | 57.0 | 2.6e-08 |
| ACB47083.1 | putative\_ABC\_transporter\_ATP-binding\_protein | BGC0001060 | Polyketide | 28.0 | 83.0 | 57.0 | 2.6e-08 |
| CCA53800.1 | putative\_ABC\_transporter | BGC0001801 | NRP | 26.0 | 90.3 | 57.0 | 2.6e-08 |
| CAG25756.1 | putative\_ABC\_transporter\_ATP-binding\_protein | BGC0000311 | NRP | 28.0 | 92.7 | 57.0 | 3.5e-08 |
| AAU34217.1 | ABC\_transporter\_MppX | BGC0000388 | NRP | 26.0 | 95.5 | 57.0 | 3.5e-08 |
| BAB08163.1 | SrtT | BGC0000556 | RiPP:Lanthipeptide | 25.0 | 99.6 | 57.0 | 4.5e-08 |
| CAG34715.1 | putative\_ABC\_transporter | BGC0000713 | Saccharide | 22.0 | 91.1 | 57.0 | 4.5e-08 |
| ALV82359.1 | ABC\_transporter\_ATP-binding\_protein | BGC0001370 | NRP | 26.0 | 89.9 | 57.0 | 4.5e-08 |
| AAM80540.1 | StaU | BGC0000290 | NRP:Glycopeptide | 26.0 | 92.7 | 56.0 | 5.9e-08 |
| ACR50788.1 | putative\_ABC\_transporter,\_ATP\_binding\_component | BGC0000163 | Polyketide | 29.0 | 88.3 | 56.0 | 7.7e-08 |
| ACR33057.1 | ABC\_transporter | BGC0000495 | RiPP:Lanthipeptide | 26.0 | 87.4 | 56.0 | 7.7e-08 |
| BAC87909.1 | probable\_ABC\_transporter | BGC0000294 | NRP | 26.0 | 88.7 | 56.0 | 1e-07 |
| AGM14936.1 | MacB-like\_ABC\_transporter\_protein | BGC0000463 | NRP:Lipopeptide | 26.0 | 87.9 | 56.0 | 1e-07 |
| BAD99411.1 | hypothetical\_protein | BGC0000634 | Terpene | 27.0 | 102.0 | 56.0 | 1e-07 |
| CAF32377.1 | putative\_ABC\_transporter,\_ATP-binding\_protein | BGC0000712 | Saccharide | 25.0 | 90.7 | 56.0 | 1e-07 |
| AAS55714.1 | ATP-binding\_protein | BGC0000795 | Saccharide | 24.0 | 86.2 | 56.0 | 1e-07 |
| WP\_019032750.1 | multidrug\_ABC\_transporter\_ATP-binding\_protein | BGC0001331 | NRP:Cyclic depsipeptide + Polyketide:Modular type I | 28.0 | 85.8 | 56.0 | 1e-07 |
| OLZ52458.1 | ABC\_transporter | BGC0001462 | NRP:Glycopeptide | 28.0 | 91.1 | 56.0 | 1e-07 |
| BAI23328.1 | putative\_ABC-transporter | BGC0000949 | NRP | 27.0 | 91.5 | 55.0 | 1.3e-07 |
| CAB38595.1 | putative\_ABC\_transporter\_ATP-binding\_protein | BGC0000315 | NRP:Ca+-dependent lipopeptide | 25.0 | 89.5 | 55.0 | 1.7e-07 |
| AAO56331.1 | syringafactin\_efflux\_protein\_SyfD | BGC0000435 | NRP | 27.0 | 88.7 | 55.0 | 1.7e-07 |
| AHI59106.1 | putative\_ATP\_binding\_protein\_of\_ABC\_transporter | BGC0001005 | NRP + Polyketide | 24.0 | 93.9 | 55.0 | 1.7e-07 |
| orf2 | multidrug\_ABC\_transporter\_ATP-binding\_protein | BGC0001422 | NRP:Cyclic depsipeptide + Polyketide:Iterative type I | 32.0 | 58.3 | 54.0 | 3.8e-07 |
| APZ78724.1 | multidrug\_ABC\_transporter\_ATP-binding\_protein | BGC0001421 | NRP:Cyclic depsipeptide + Polyketide:Iterative type I | 32.0 | 58.3 | 55.0 | 1.7e-07 |
| APZ78751.1 | multidrug\_ABC\_transporter\_ATP-binding\_protein | BGC0001423 | NRP:Cyclic depsipeptide + Polyketide:Iterative type I | 33.0 | 58.3 | 55.0 | 1.7e-07 |
| YP\_002539287.1 | IroC,\_ABC\_transport\_protein | BGC0001555 | RiPP | 23.0 | 91.9 | 55.0 | 1.7e-07 |
| CCQ18690.1 | ABC\_transporter\_ATP-binding\_protein | BGC0000544 | RiPP:Lanthipeptide | 28.0 | 67.2 | 54.0 | 2.2e-07 |
| BAB78444.1 |  | BGC0000555 | RiPP:Lanthipeptide | 24.0 | 82.2 | 54.0 | 2.2e-07 |
| ACN80635.1 | Sio4 | BGC0000655 | Terpene | 24.0 | 86.6 | 54.0 | 2.2e-07 |
| WP\_047890617.1 | ABC\_transporter\_ATP-binding\_protein | BGC0001330 | NRP:Cyclic depsipeptide + Polyketide:Modular type I | 26.0 | 85.0 | 54.0 | 2.2e-07 |
| ATY72532.1 | ABC\_transporter\_ATP-binding\_protein | BGC0001574 | NRP | 29.0 | 75.3 | 54.0 | 2.2e-07 |
| AEZ53959.1 | putative\_ABC\_transporter\_ATP-binding\_protein | BGC0000144 | Polyketide:Modular type I | 28.0 | 87.4 | 54.0 | 3.8e-07 |
| APZ78737.1 | multidrug\_ABC\_transporter\_ATP-binding\_protein | BGC0001422 | NRP:Cyclic depsipeptide + Polyketide:Iterative type I | 32.0 | 58.3 | 54.0 | 3.8e-07 |
| WP\_078957969.1 | ABC\_transporter\_ATP-binding\_protein | BGC0001551 | RiPP | 28.0 | 91.1 | 53.0 | 5e-07 |
| CAF33315.1 | putative\_ABC\_transporter,\_ATP-binding\_protein,\_NeoU | BGC0000709 | Saccharide | 21.0 | 91.1 | 52.0 | 8.5e-07 |
| CAH58693.1 | putative\_exporter | BGC0000711 | Saccharide | 21.0 | 91.1 | 52.0 | 8.5e-07 |
| AJE44468.1 | hypothetical\_protein | BGC0001779 | RiPP | 26.0 | 89.1 | 52.0 | 8.5e-07 |
| WP\_122215939.1 | ATP-binding\_cassette\_domain-containing\_protein | BGC0002015 | RiPP | 25.0 | 87.4 | 52.0 | 8.5e-07 |
| EHN79455.1 | ABC\_transporter\_ATP-binding\_protein\_ScatW | BGC0000804 | Saccharide | 24.0 | 89.9 | 52.0 | 1.1e-06 |
| OJF16281.1 | AceT2 | BGC0001491 | Polyketide | 24.0 | 92.7 | 52.0 | 1.1e-06 |
| SFF02632.1 | ABC-2\_type\_transport\_system\_ATP-binding\_protein | BGC0001674 | RiPP | 24.0 | 87.9 | 52.0 | 1.1e-06 |
| BAJ19059.1 | putative\_ABC\_transporter | BGC0000288 | NRP | 25.0 | 99.6 | 52.0 | 1.4e-06 |
| EHQ60564.1 | ABC\_transporter | BGC0001356 | RiPP | 25.0 | 95.5 | 52.0 | 1.4e-06 |
| ANQ31705.1 | ABC\_transporter | BGC0001395 | Other | 37.0 | 35.2 | 52.0 | 1.4e-06 |
| AQZ42156.1 | putative\_ABC\_multidrug\_transporter | BGC0001820 | NRP | 24.0 | 93.9 | 52.0 | 1.4e-06 |
| CAM56766.1 | ExpB | BGC0000354 | NRP | 22.0 | 87.0 | 51.0 | 1.9e-06 |
| QCC62989.1 | hypothetical\_protein | BGC0001966 | NRP | 23.0 | 89.1 | 51.0 | 1.9e-06 |
| BAJ05895.1 | putative\_ABC\_multidrug\_resistance\_transporter | BGC0000872 | Other | 27.0 | 92.7 | 50.0 | 3.2e-06 |
| ORC16572.1 | hypothetical\_protein | BGC0001341 | NRP | 26.0 | 78.5 | 50.0 | 3.2e-06 |
| BBD82038.1 | ABC\_transporter | BGC0001921 | Other | 24.0 | 88.3 | 50.0 | 3.2e-06 |
| ALD83689.1 | ATP\_binding\_protein | BGC0001300 | Polyketide | 27.0 | 79.8 | 50.0 | 4.2e-06 |
| ABD14713.1 | CesC | BGC0000320 | NRP:Cyclic depsipeptide | 24.0 | 88.7 | 49.0 | 7.2e-06 |
| ABC50111.1 | unknown | BGC0000643 | Terpene | 25.0 | 87.0 | 49.0 | 7.2e-06 |
| BAQ25488.1 | multidrug\_ABC\_transporter\_ATP-binding\_protein | BGC0001288 | Polyketide | 25.0 | 84.6 | 49.0 | 7.2e-06 |
| WP\_053138477.1 | ABC-F\_type\_ribosomal\_protection\_protein\_Srm(B) | BGC0002033 | Polyketide | 27.0 | 68.8 | 49.0 | 7.2e-06 |
| AFV25467.1 | hypothetical\_protein | BGC0000469 | RiPP:Bottromycin | 22.0 | 92.7 | 49.0 | 9.4e-06 |
| CBW74823.1 | Multidrug\_resistance\_ABC\_transporter\_ATP-binding\_and\_permease\_protein | BGC0000571 | RiPP:Lassopeptide | 23.0 | 93.9 | 49.0 | 9.4e-06 |
| AAN85531.1 | ABC\_transporter\_component,\_ATP\_hydrolase | BGC0001101 | NRP + Polyketide:Modular type I + Polyketide:Trans-AT type I | 24.0 | 94.7 | 49.0 | 9.4e-06 |
| BAP82652.1 | ABC\_transporter | BGC0001148 | NRP + RiPP | 25.0 | 97.2 | 49.0 | 9.4e-06 |
| WP\_078620925.1 | ABC\_transporter\_ATP-binding\_protein | BGC0001549 | RiPP | 29.0 | 64.4 | 49.0 | 9.4e-06 |
