# Supplementary material for: Genome of Methylomonas sp. AM2-LC, representing a methanotrophic bacterial species isolated from water column of a boreal, oxygen-stratified lake
Source: Front Genet. 2024 Aug 30;15:1440435. doi: 10.3389/fgene.2024.1440435 (PMC11392852; doi:10.3389/fgene.2024.1440435)
Supplement: Supplementary file 3 [file DataSheet2.ZIP › Supplementary_File_2/08.gene_cluster/AMSG_genome.zip!/knownclusterblast/region6/GE004207_mibig_hits.html]

| MIBiG Protein | Description | MIBiG Cluster | MiBiG Product | % ID | % Coverage | BLAST Score | E-value |
| --- | --- | --- | --- | --- | --- | --- | --- |
| ABU41511.1 | HemB | BGC0000906 | Other | 53.0 | 97.0 | 325.0 | 1.2e-88 |
| ABT18045.1 | delta-aminolevulinic\_acid\_dehydrotase/porphobilinogen\_synthase | BGC0000905 | Other | 50.0 | 96.7 | 312.0 | 8.1e-85 |
| EHT12134.1 | delta-aminolevulinic\_acid\_dehydratase | BGC0001685 | Other | 44.0 | 95.2 | 242.0 | 1e-63 |
