# Supplementary material for: Genome of Methylomonas sp. AM2-LC, representing a methanotrophic bacterial species isolated from water column of a boreal, oxygen-stratified lake
Source: Front Genet. 2024 Aug 30;15:1440435. doi: 10.3389/fgene.2024.1440435 (PMC11392852; doi:10.3389/fgene.2024.1440435)
Supplement: Supplementary file 3 [file DataSheet2.ZIP › Supplementary_File_2/08.gene_cluster/AMSG_genome.zip!/knownclusterblast/region6/GE004197_mibig_hits.html]

| MIBiG Protein | Description | MIBiG Cluster | MiBiG Product | % ID | % Coverage | BLAST Score | E-value |
| --- | --- | --- | --- | --- | --- | --- | --- |
| ADQ55487.1 | peptidase\_U62 | BGC0000350 | NRP:Beta-lactam | 50.0 | 100.6 | 473.0 | 3.9e-133 |
| ADB92557.1 | CcbIH | BGC0001225 | NRP | 30.0 | 44.9 | 91.0 | 4.2e-18 |
| ABX00605.1 | LmbIH | BGC0000907 | Other | 32.0 | 45.9 | 89.0 | 2.1e-17 |
