# Supplementary material for: Genome of Methylomonas sp. AM2-LC, representing a methanotrophic bacterial species isolated from water column of a boreal, oxygen-stratified lake
Source: Front Genet. 2024 Aug 30;15:1440435. doi: 10.3389/fgene.2024.1440435 (PMC11392852; doi:10.3389/fgene.2024.1440435)
Supplement: Supplementary file 3 [file DataSheet2.ZIP › Supplementary_File_2/08.gene_cluster/AMSG_genome.zip!/knownclusterblast/region6/GE004201_mibig_hits.html]

| MIBiG Protein | Description | MIBiG Cluster | MiBiG Product | % ID | % Coverage | BLAST Score | E-value |
| --- | --- | --- | --- | --- | --- | --- | --- |
| BAC76462.1 | pyrroloquinoline\_quinone\_biosynthesis\_protein\_B | BGC0001100 | NRP + Polyketide | 37.0 | 99.0 | 196.0 | 7.6e-50 |
