# Supplementary material for: Genome of Methylomonas sp. AM2-LC, representing a methanotrophic bacterial species isolated from water column of a boreal, oxygen-stratified lake
Source: Front Genet. 2024 Aug 30;15:1440435. doi: 10.3389/fgene.2024.1440435 (PMC11392852; doi:10.3389/fgene.2024.1440435)
Supplement: Supplementary file 3 [file DataSheet2.ZIP › Supplementary_File_2/08.gene_cluster/AMSG_genome.zip!/knownclusterblast/region6/GE004204_mibig_hits.html]

| MIBiG Protein | Description | MIBiG Cluster | MiBiG Product | % ID | % Coverage | BLAST Score | E-value |
| --- | --- | --- | --- | --- | --- | --- | --- |
| BAC76463.1 | pyrroloquinoline\_quinone\_biosynthesis\_protein\_E | BGC0001100 | NRP + Polyketide | 47.0 | 90.1 | 347.0 | 2.5e-95 |
| ABT18046.1 | heme\_d1\_biosynthesis\_protein | BGC0000905 | Other | 25.0 | 88.5 | 86.0 | 8e-17 |
| WP\_019503880.1 | nif11-class\_peptide\_radical\_SAM\_maturase\_3 | BGC0001745 | RiPP | 25.0 | 90.4 | 84.0 | 5.2e-16 |
| ABU41512.1 | NirJ2 | BGC0000906 | Other | 25.0 | 89.0 | 80.0 | 7.5e-15 |
| WP\_015143117.1 | nif11-class\_peptide\_radical\_SAM\_maturase\_3 | BGC0001733 | RiPP | 24.0 | 87.7 | 77.0 | 3.7e-14 |
| ABU41510.1 | NirJ1 | BGC0000906 | Other | 29.0 | 42.2 | 66.0 | 1.5e-10 |
| CAB11985.2 | synthesis\_of\_sporulation\_killing\_factor\_A | BGC0000601 | RiPP:Head-to-tailcyclized peptide | 24.0 | 48.4 | 62.0 | 2.1e-09 |
| ABT18044.1 | heme\_d1\_biosynthesis\_protein | BGC0000905 | Other | 31.0 | 30.5 | 55.0 | 2e-07 |
