# Supplementary material for: Genome of Methylomonas sp. AM2-LC, representing a methanotrophic bacterial species isolated from water column of a boreal, oxygen-stratified lake
Source: Front Genet. 2024 Aug 30;15:1440435. doi: 10.3389/fgene.2024.1440435 (PMC11392852; doi:10.3389/fgene.2024.1440435)
Supplement: Supplementary file 3 [file DataSheet2.ZIP › Supplementary_File_2/08.gene_cluster/AMSG_genome.zip!/knownclusterblast/region6/GE004193_mibig_hits.html]

| MIBiG Protein | Description | MIBiG Cluster | MiBiG Product | % ID | % Coverage | BLAST Score | E-value |
| --- | --- | --- | --- | --- | --- | --- | --- |
| AKD43750.1 | HerI | BGC0001349 | NRP + Polyketide | 26.0 | 96.2 | 103.0 | 8.1e-22 |
| ACO94485.1 | glycine\_oxidase/FAD-dependent\_oxidoreductase | BGC0000097 | Polyketide:Modular type I | 26.0 | 98.9 | 102.0 | 1.4e-21 |
| BAO66527.1 | FAD-dependent\_glycine\_oxydase | BGC0000042 | Polyketide | 27.0 | 103.3 | 99.0 | 1.2e-20 |
| ACO94457.1 | glycine\_oxidase/FAD-dependent\_oxidoreductase | BGC0000029 | Polyketide:Modular type I | 26.0 | 98.9 | 88.0 | 2.7e-17 |
