# Supplementary material for: Genome of Methylomonas sp. AM2-LC, representing a methanotrophic bacterial species isolated from water column of a boreal, oxygen-stratified lake
Source: Front Genet. 2024 Aug 30;15:1440435. doi: 10.3389/fgene.2024.1440435 (PMC11392852; doi:10.3389/fgene.2024.1440435)
Supplement: Supplementary file 3 [file DataSheet2.ZIP › Supplementary_File_2/08.gene_cluster/AMSG_genome.zip!/knownclusterblast/region6/GE004206_mibig_hits.html]

| MIBiG Protein | Description | MIBiG Cluster | MiBiG Product | % ID | % Coverage | BLAST Score | E-value |
| --- | --- | --- | --- | --- | --- | --- | --- |
| ABU70358.1 | hypothetical\_protein | BGC0001890 | NRP | 39.0 | 97.4 | 235.0 | 1.3e-61 |
| ABU70359.1 | hypothetical\_protein | BGC0001890 | NRP | 39.0 | 97.4 | 235.0 | 1.3e-61 |
| CBJ79892.1 | transposase | BGC0001133 | NRP | 39.0 | 96.2 | 228.0 | 2.1e-59 |
| RALTA\_B1233 | Transposase\_fragment,\_IS1111\_family | BGC0001189 | NRP | 38.0 | 68.9 | 146.0 | 7.9e-35 |
